# Reviewer acknowledgement 2014

**DOI:** 10.1186/s12889-015-1368-3

**Published:** 2015-02-14

**Authors:** Natalie Pafitis

**Affiliations:** Executive Editor, BMC Public Health, London, UK

**Jens Aagaard-Hansen**

Denmark

**Geir Aamodt**

Norway

**Pauliina Aarnio**

Finland

**Said Abbadi**

Egypt

**Bayad Abdalrahman**

UK

**Anees Ahmed Abdul Pari**

UK

**Abu Saleh Abdullah**

USA

**Abu Abdul-Quader**

USA

**Sawsan Abdulrahim**

Lebanon

**Fantu Abebe**

Ethiopia

**Gemeda Abebe**

Ethiopia

**Femke Abma**

Netherlands

**Frances Aboud**

Canada

**Mauro Henrique Abreu**

Brazil

**Sandra Abreu**

Portugal

**Clemente Abrokwaa**

USA

**Karim Abu-Omar**

Germany

**Chandran Achutan**

USA

**Ishag Adam**

Sudan

**Philippe Adam**

Australia

**Kathryn Adams**

USA

**Marc Adams**

USA

**Richard Adanu**

Ghana

**Adedeji Adefuye**

USA

**Adetunji Oladeni Adeniji**

Nigeria

**Ifedayo Adetifa**

Kenya

**Ramesh Adhikari**

Nepal

**Rajatashuvra Adhikary**

USA

**Richard Adome**

Uganda

**Wichai Aekplakorn**

Thailand

**Israel Agaku**

Nigeria

**Keren Agay-Shay**

Spain

**DeBrenna Agbenyiga**

USA

**Pakhee Aggarwal**

India

**Paul Agius**

Australia

**Kiran Agrahari**

India

**Charles Agyemang**

Netherlands

**Anand Ahankari**

UK

**Sk. Akhtar Ahmad**

Bangladesh

**Zeeshan Ahmed**

Pakistan

**Ismael Ahmed**

Ethiopia

**Munir Ahmed**

Canada

**Naheed Ahmed**

USA

**Kirsi Ahola**

Finland

**Aima Ahonkhai**

USA

**Jun Aida**

UK

**Allison Aiello**

USA

**Barbara Ainsworth**

USA

**Marja Airaksinen**

Finland

**Luísa Aires**

Portugal

**Akseli Aittomäki**

Finland

**Yoshifusa Aizawa**

Japan

**Olusegun Ajenifuja**

Nigeria

**Sally Akarolo-Anthony**

USA

**Laila Akhu-Zaheya**

Jordan

**Dora Olufunmilola Akinboye**

Nigeria

**Manas Akmatov**

Germany

**Christopher Akolo**

USA

**Shamima Akter**

Japan

**Khalid Al Rasadi**

Oman

**Haleama Al Sabbah**

United Arab Emirates

**Olufunke Alaba**

South Africa

**Khurshed Alam**

Bangladesh

**Noore Alam**

Australia

**Steven Albert**

USA

**Sami Al-Dubai**

Malaysia

**Tadesse Alemayehu**

Ethiopia

**Sheila Alessi**

USA

**Evangelos Alexopoulos**

Greece

**Suliman Alghnam**

Saudi Arabia

**Paul Allanson**

UK

**Elias Allara**

Italy

**Stephanie Alley**

Australia

**Hesham Al-Mekhlafi**

Malaysia

**Suzana Almoosawi**

UK

**Lena Almqvist**

Sweden

**Redhwan Al-Naggar**

Malaysia

**Harith Al-Qazaz**

Malaysia

**Ayman Alsulaiman**

Saudi Arabia

**Jose Luis Alvarez**

UK

**Gonzalo G. Alvarez**

Canada

**Alberto Alves**

Portugal

**Luana Alves**

Brazil

**Veronica Alves**

Brazil

**Heba Alwan**

Switzerland

**Eliana Amaral**

Brazil

**Maria Luiza Amaral**

Brazil

**Argaw Ambelu**

Ethiopia

**Alemayehu Amberbir**

Malawi

**Shin Amemiya**

Japan

**Shanthi Ameratunga**

New Zealand

**Daniela Amicizia**

Italy

**Hugo Amigo**

Chile

**Tarek Amin**

Saudi Arabia

**Sedigheh Amini Kafi-abad**

Iran

**Nayyereh Aminisani**

Australia

**Yuri Amirkhanian**

USA

**Noh Amit**

Malaysia

**Emanuele Amodio**

Italy

**Olukemi Amodu**

Nigeria

**Olorunfemi Amoran**

Nigeria

**Matthias An der Heiden**

Germany

**Aase Bengaard Andersen**

Denmark

**Ingelise Andersen**

Denmark

**Benjamin Anderson**

USA

**Rob Anderson**

UK

**Sarah Anderson**

USA

**Sigmund Anderssen**

Norway

**Hege K. Andreassen**

Norway

**Gabriel Andreuccetti**

Brazil

**Nadine Andrew**

Australia

**Jason Andrews**

USA

**Monica Andrews**

Chile

**Robert Andrews**

UK

**Katherine Andrinopoulos**

USA

**Jon Kim Andrus**

USA

**Johannes R Anema**

Netherlands

**Siddhartha Angadi**

USA

**Juana Angel**

Colombia

**Sara Angleman**

Sweden

**Worku Animaw**

Ethiopia

**Ranjit Mohan Anjana**

India

**Matilda Annerstedt**

Sweden

**Donna Ansara**

USA

**Nicola Ansell**

UK

**Magda Antal**

Hungary

**Aderaw Jose Leopoldo Anteneh**

Ethiopia

**Ferreira Antunes**

Brazil

**Rose Apondi**

Uganda

**Nuria Aragonés**

Spain

**Hirokazu Arai**

Japan

**Margaret Araoye**

Nigeria

**Chrisa Arcan**

USA

**Edward Archer**

USA

**Chris Archibald**

Canada

**Thiago Ardenghi**

Brazil

**Paul Arguin**

USA

**Jorge Arias**

USA

**Elva Dolores Arias-Merino**

Mexico

**Caroline Ariën**

Belgium

**Inci Arikan**

Turkey

**Hisatomi Arima**

Japan

**Juan C Aristizabal**

Colombia

**Jason Armfield**

Australia

**Nicolas Arnaud**

Germany

**Bengt Arnetz**

USA

**Lisen Arnheim Dahlström**

Sweden

**Benjamin Arnold**

USA

**Arpo Aromaa**

Finland

**Britt Arrelov**

Sweden

**Silvina Arrossi**

Argentina

**Benoit Arsenault**

Netherlands

**Didem Arslantas**

Turkey

**Takashi Asakura**

Japan

**Mulugeta Aseratie**

Ethiopia

**Bryndis Bjork Asgeirsdottir**

Iceland

**Richard Ashcroft**

UK

**Tigistu Adamu Ashengo**

USA

**Mark Ashworth**

UK

**Kingsley Asiedu**

Switzerland

**Ian Askew**

Kenya

**Roland Asmar**

France

**Thor Aspelund**

Iceland

**Esther Aspinall**

UK

**Peter John Aspinall**

UK

**Sawitri Assanangkornchai**

Thailand

**Shervin Assari**

USA

**Nega Assefa**

Ethiopia

**Yibeltal Assefa**

Ethiopia

**Feleke Astawesegn**

Ethiopia

**Melinda Asztalos**

Belgium

**Vasilios Athyros**

Greece

**Andrew J Atkin**

UK

**Hani Atrash**

USA

**Amir Attaran**

Canada

**Lukoye Atwoli**

Kenya

**Tim Aubry**

Canada

**Erik Augustson**

USA

**Robert Aunger**

UK

**Aine Aventin**

UK

**Rukman Awang Hamat**

Malaysia

**Niyi Awofeso**

Australia

**Olutosin Alaba Awolude**

Nigeria

**David Awonuga**

Nigeria

**Ayodeji Awopegba**

USA

**Olalekan Ayo-Yusuf**

South Africa

**Darshini Ayton**

Australia

**Mohammad Azam**

Australia

**Luis F Azevedo**

Portugal

**Syed Khurram Azmat**

Canada

**Clement C. Azodo**

Nigeria

**Peter Baade**

Australia

**Adriana Baban**

Romania

**Chantal Babb**

South Africa

**Damodar Bachani**

India

**Amaia Bacigalupe**

Spain

**Hannah Badland**

Australia

**Stéphanie Baggio**

Switzerland

**Aaron Baggish**

USA

**Prue Bagley**

USA

**Anne-Marie Bagnall**

UK

**Chyi-Huey Bai**

Taiwan

**Julia Bailey**

UK

**Olusegun Baiyewu**

Nigeria

**Anna Bajer**

Poland

**Sabrina Bakeera-Kitaka**

Uganda

**Chris Baker**

UK

**Graham Baker**

UK

**Inger Johanne Bakken**

Norway

**Arnold Bakker**

Netherlands

**Ioannis Bakolis**

UK

**James Balcius**

USA

**Alberto Baldasseroni**

Italy

**Vincenzo Baldo**

Italy

**Elizabeth Ballard**

USA

**Eric Balti**

Belgium

**James Bamidele**

Nigeria

**Jorge Banda**

USA

**Mohammad Reza Baneshi**

Iran

**Wei Bao**

USA

**Negga Baraki**

Ethiopia

**Gianvincenzo Barba**

Italy

**Pamela Barbadoro**

Italy

**Lindley Barbee**

USA

**Mary Barker**

UK

**Emily Barnes**

USA

**Simon Barquera**

Mexico

**Leila Barraj**

USA

**Sean Barrett**

Canada

**Aluisio J D Barros**

Brazil

**Peter Barss**

Canada

**Bernadette Bartlam**

UK

**Rashid Bashshur**

USA

**Tibor Baska**

Slovakia

**David Bassett**

USA

**Paulo Basta**

Brazil

**Tânia Bastos**

Portugal

**Eric Bateman**

South Africa

**John Batsis**

USA

**Lesley Batten**

New Zealand

**Greta Bauer**

Canada

**Michele Baumann**

Luxembourg

**Sebastian Baumeister**

Germany

**Amanda J Baxter**

Australia

**Alison Beauchamp**

Australia

**Nancy Beauregard**

Canada

**Stevens Bechange**

Uganda

**Elisardo Becoña**

Spain

**Malgorzata Bednarska**

Poland

**Stephen Begg**

Australia

**Andrea Begley**

Australia

**Wang Bei**

China

**Ulla Beijer**

Sweden

**Tinneke Beirens**

Netherlands

**Tefera Belachew**

Ethiopia

**Leo Beletsky**

USA

**Vicente Jr. Belizario**

Philippines

**Robin Bell**

Australia

**Steven Bell**

UK

**Silvia Bel-Serrat**

Spain

**Daniel Belsky**

USA

**Ben Belton**

Bangladesh

**Yeshambel Belyhun**

Ethiopia

**Ahmed Ben Abdelaziz**

Tunisia

**Ziyad Ben Taleb**

USA

**Sandra Benavides**

USA

**Christine Benn**

Denmark

**Abdelouaheb Bennani**

Morocco

**David Bennett**

USA

**Janie Benson**

USA

**Tony Benson**

UK

**Tanya Beran**

Canada

**Sarah Berdot**

France

**Janneke Berecki-Gisolf**

Australia

**Sverre Bergh**

Norway

**Erik Berglund**

Sweden

**Patrick Bergman**

Sweden

**Gunnar Bergström**

Sweden

**Yemane Berhane**

Ethiopia

**Gebretsadik Berhe**

Ethiopia

**Anita Berlin**

Sweden

**Autumn Bernal**

USA

**Sveinung Berntsen**

Norway

**Diane Berry**

USA

**Chiara Bertoncello**

Italy

**Elizabeth Bertone-Johnson**

USA

**Kathelijne Bessems**

Netherlands

**Pascal Bessong**

South Africa

**Jane Bethea**

UK

**Elizabeth Beverly**

USA

**Getenet Beyene**

Ethiopia

**Nulda Beyers**

South Africa

**Nicholas Beyler**

USA

**Kavi Bhalla**

USA

**Ruchi Bhandari**

USA

**Miteshkumar Bhanderi**

India

**Aradhana Bhargava**

India

**Madhav Bhatta**

USA

**Maitree Bhattacharyya**

India

**Upendra Bhojani**

India

**Kamaldeep Bhui**

UK

**Abbas Bhuiya**

Bangladesh

**Zulfiqar Bhutta**

Pakistan

**Sibhatu Biadgilign**

Ethiopia

**Silvia Bianchi**

Italy

**Matt Bianchi**

USA

**Jerome Bickenbach**

Switzerland

**Steve Bickler**

USA

**Satesh Bidaisee**

Grenada

**Stuart Biddle**

UK

**Ettore Bidoli**

Italy

**Renata Bielemann**

Brazil

**Robin Biellik**

UK

**Damir Bikmukhametov**

Netherlands

**Jade Bilardi**

Australia

**Nazan Bilgel**

Turkey

**Daniel Bingham**

UK

**Samantha Birch**

UK

**Marie-Louise Bird**

Australia

**Michelle Birkett**

USA

**Wendy Birmingham**

USA

**Richard Birtwhistle**

Canada

**Zeno Bisoffi**

Italy

**Masa Bizjak Cernelic**

Slovenia

**Mona Bjelland**

Norway

**Tony Blakely**

New Zealand

**Francois-Xavier Blanc**

France

**Dianna Blau**

USA

**Evan Blecher**

USA

**Hannah Blencowe**

UK

**Ilse Blignault**

Australia

**Renette Blignaut**

South Africa

**Jenny Bliltvich**

Australia

**Jonathan Blitstein**

USA

**Valarie Blue Bird Jernigan**

USA

**Ricky Bluthenthal**

USA

**Martin Bobak**

UK

**Petri Böckerman**

Finland

**Said Bodur**

Turkey

**Alexandria Boehm**

USA

**Inge Bogaert**

Belgium

**Ilze Bogdanovica**

UK

**Barry Bogin**

UK

**Lucimere Bohn**

Portugal

**Malin Bolin**

Sweden

**Catherine Bolman**

Netherlands

**Chris Bonell**

UK

**Matteo Bonzini**

Italy

**Mei Ying Boon**

Australia

**Cecile RL Boot**

Netherlands

**Helen Booth**

UK

**Kristin Benjaminsen Borch**

Norway

**Michael Borg**

Malta

**Michael Borghese**

Canada

**Ron Borland**

Australia

**Betty Borowsky**

USA

**Annet Bosch**

Netherlands

**Julitta Boschman**

Netherlands

**Jizzo Bosdriesz**

Netherlands

**William Bosu**

Burkina Faso

**Sarah Bott**

USA

**Abderrahmane Boudoukha**

Algeria

**Sofia Bouhlal**

USA

**Lucy Boulanger**

USA

**Stephen Bouquin**

France

**Adelia Bovell-Benjamin**

USA

**Deborah Bowen**

USA

**Natasha Bowen**

USA

**Anders Boyd**

France

**William Boyer**

USA

**Allison Boyes**

Australia

**Elaine Boyle**

UK

**Murat Boysan**

Turkey

**Ivana Bozicevic**

Croatia

**Marek Brabec**

Czech Republic

**Nicola Luigi Bragazzi**

Italy

**Paula Braitstein**

USA

**Anke Bramesfeld**

Germany

**Tilman Brand**

Germany

**Serge Brand**

Switzerland

**James Brandful**

Ghana

**Phillip Brantley J**

USA

**Amy Branum**

USA

**John Bratt**

USA

**Julia Braun**

Switzerland

**Annette Joy Braunack-Mayer**

Australia

**João Breda**

Denmark

**Amin Bredan**

Belgium

**Elizabeth Breeze**

UK

**Jean Brender**

USA

**Aline Brennan**

Ireland

**Thomas Brett**

Australia

**Christian Brettschneider**

Germany

**Roberto Briceño-León**

Venezuela

**William Brieger**

USA

**Frederic Briere**

Canada

**Julie Brimblecombe**

Australia

**Erica Briones-Vozmediano**

Spain

**Carina Sjöberg Brixval**

Denmark

**Paula Brochu**

USA

**David Brock**

USA

**Thomas Brockamp**

Germany

**Elizabeth Brondolo**

USA

**Henrik Bronnum-Hansen**

Denmark

**Katherine Brooke-Wavell**

UK

**Richard Brostrom**

USA

**Amanda Brouwer**

USA

**Monique Brown**

USA

**Katherine Brown**

UK

**Tamara Brown**

UK

**Johannes Brug**

Netherlands

**Tim Brusseau**

USA

**Olivier Bruyère**

Belgium

**Robert Brychta**

USA

**Susan Buchbinder**

USA

**Maciej Buchowski**

USA

**John Buckley**

UK

**Jens Bucksch**

Germany

**Lorraine Buis**

USA

**Yves Buisson**

Laos

**Susan Bull**

UK

**David Buller**

USA

**Monika Bullinger**

Germany

**Caroline Bulsara**

Australia

**Maria Bulzacchelli**

USA

**Matthew Buman**

USA

**Ruveyde Bundak**

Turkey

**Alex Burdorf**

Netherlands

**Clara Burgert**

USA

**Duika Burges Watson**

UK

**Rachel Burke**

USA

**Shauna Burke**

Canada

**Gregor Burkhart**

Portugal

**Howard Burkom**

USA

**Janet Burnett**

USA

**Jacob Burns**

Germany

**Jan Burns**

UK

**Luca Busani**

Italy

**Vincent Busch**

Netherlands

**Hermann Bussmann**

Botswana

**Joanna Busza**

UK

**India Butler**

South Africa

**Lisa Butler**

USA

**Tony Butler**

Australia

**Alison Buttenheim**

USA

**Jane A Buxton**

Canada

**Joanne Buzaglo**

USA

**Llorenç Caballeria**

Spain

**Francisco Felix Caballero**

Spain

**Noriko Cable**

UK

**Antonio Cabral**

Portugal

**Amaia Calderón-Larrañaga**

Spain

**Alison Calear**

Australia

**Anna Callan**

Australia

**Denton Callander**

Australia

**Guido Calleri**

Italy

**Robin Callister**

Australia

**Simona Calugi**

Italy

**Emmanuelle Cambois**

France

**Adrian Cameron**

Australia

**Laura Camoni**

Italy

**Christine Campbell**

UK

**Ahmet Selçuk Can**

Turkey

**Maureen Canavan**

USA

**Stefano Candura**

Italy

**Karen Canfell**

Australia

**Chunxiang Cao**

China

**Cristina Caperchione**

Canada

**Paolo Capodaglio**

Italy

**Pasquale Caponnetto**

Italy

**Peter Caputi**

Australia

**Marly Cardoso**

Brazil

**Gemma Carey**

Australia

**Rachel Carey**

Australia

**Shannon Carlin-Menter**

USA

**Susan Carnell**

UK

**Christin Carotta**

USA

**Belinda Carpenter**

Australia

**Giulia Carreras**

Italy

**Owen Carter**

Australia

**Maria Joana Carvalho**

Portugal

**Andreia Cascaes**

Brazil

**Patrick Cashman**

Australia

**Annalisa Casini**

Belgium

**Carlos Castañeda-Orjuela**

Colombia

**Darla Castelli**

USA

**Katia Castetbon**

France

**Alvaro Castillo-Carniglia**

Chile

**Lilia Castillo-Martinez**

Mexico

**Jessica Castonguay**

USA

**Enrique Castro-Sánchez**

UK

**Caricia Catalani**

USA

**Carlo Catassi**

Italy

**Dolores Catelan**

Italy

**Delwyn Catley**

USA

**Gillian Caughey**

Australia

**Franco Cavallo**

Italy

**Nick Cavill**

UK

**Joan A. Caylà**

Spain

**Gülcan Çetin**

Turkey

**Amit Chakrabarti**

India

**Nilanjan Chakraborty**

India

**Venkatesan Chakrapani**

India

**Kirsty Challen**

UK

**Mohammadou Kabir Cham**

Gambia

**Catherine Chamberlain**

Australia

**Charlotte Chamberlain**

UK

**Gabriel Chamie**

USA

**Geoffrey Chan**

Australia

**Pierre Chandon**

France

**Mei-Wei Chang**

USA

**Larry Chang**

USA

**Victor Chang**

USA

**M Pia Chaparro**

Sweden

**Man Charurat**

USA

**Charles Chasela**

South Africa

**Kiron Chatterjee**

UK

**Allen Cheadle**

USA

**William Checkley**

USA

**Rima Chedid**

Lebanon

**Elisabetta Chellini**

Italy

**Chu-Chih Chen**

Taiwan

**Chi-Ling Chen**

Taiwan

**Dafang Chen**

China

**Honghui Chen**

China

**Hong-I Chen**

Taiwan

**Jaw-Wen Chen**

Taiwan

**Szu Chieh Chen**

Taiwan

**Yu-ling Chen**

UK

**Yue Chen**

Canada

**Boon How Chew**

Malaysia

**Peilian Chi**

Macao

**Peggy Pei-Chia Chiang**

Singapore

**Alexandre Chiavegatto Filho**

Brazil

**Alain Chichom-Mefire**

Cameroon

**Chi-Wen Chien**

Australia

**Palma Chillón**

Spain

**Abdallah Chilungo**

Malawi

**Charles Chima**

USA

**Dal Lae Chin**

USA

**Deborah Chinn**

UK

**Jean-Philippe Chippaux**

Benin

**Katharine Chiseri**

USA

**Catherine Chittleborough**

Australia

**Edmond Chiu**

Australia

**Maria Chiu**

Canada

**Youngtae Cho**

Korea, South

**BongKyoo Choi**

USA

**Lillian Choi**

USA

**Herberto Jose Chong Neto**

Brazil

**Manisha Choudhury**

India

**Eric Chow**

Australia

**Hsueh-wen Chow**

Taiwan

**Gerardo Chowell**

USA

**Helen Christensen**

Australia

**Hayley Christian**

Australia

**Nicola Christie**

UK

**Karen Chronister**

Australia

**Nain-Feng Chu**

Taiwan

**Rajesh Chudasama**

India

**Daniel Church**

USA

**James Churilla**

USA

**Bruno Christian Ciancio**

Sweden

**Simon Lebech Cichosz**

Denmark

**Jose M Cisneros**

Spain

**Maria Grazia Ciufolini**

Italy

**Giovanni Cizza**

USA

**Mareli Claassens**

South Africa

**Heiner Claessen**

Germany

**Brenda Clark**

USA

**Cameron Clark**

Canada

**Jesse Clark**

USA

**Jennifer Clarke**

USA

**Bjorgulf Claussen**

Norway

**Els Clays**

Belgium

**John Cleland**

UK

**Stacy Clemes**

UK

**Dylan Cliff**

Australia

**Angela Clifford**

UK

**Jane Coad**

New Zealand

**Laura Cobb**

USA

**Frank Cobelens**

Netherlands

**Tom Cochrane**

Australia

**Bruno Coêlho**

Brazil

**Manuel Coelho e Silva**

Portugal

**Juli Coffin**

Australia

**Patience Cofie**

Ghana

**Myron Cohen**

USA

**Sarah Cohen**

USA

**Carlos E.A. Coimbra, Jr.**

Brazil

**Matthew Coldiron**

France

**Samantha Cole**

USA

**Brenda Coleman**

Canada

**Elien Colman**

Belgium

**Elizabeth Comino**

Australia

**Nicola Comodo**

Italy

**Angelo Compare**

Italy

**Elizabeth Comrie-Thomson**

Australia

**John Condon**

Australia

**Jennie Connor**

New Zealand

**Deborah Constant**

South Africa

**Susanna Conti**

Italy

**Paul Maurice Conway**

Denmark

**Won Cook**

USA

**Richard Cooke**

UK

**Peter Cooper**

South Africa

**Kristen Copeland**

USA

**Teri Copeland**

USA

**Alessandro Coppo**

Italy

**Raildo Coqueiro**

Brazil

**John Martin Corkery**

UK

**Marilia Correa da Silva**

Brazil

**Giuliana Cortese**

Italy

**Sandra Coulon**

USA

**Kristy Coxon**

Australia

**Alessandro Cozzi-Lepri**

UK

**AliceAnn Crandall**

USA

**Julian Crane**

New Zealand

**Ross Cranston**

USA

**Paul Cremers**

Netherlands

**Sue Crengle**

New Zealand

**Nicole Crepaz**

USA

**Catherine Crespi**

USA

**Maria Luisa Cristina**

Italy

**Julia Critchley**

UK

**Tim Croudace**

UK

**Rik Crutzen**

Netherlands

**Ilona Csizmadi**

Canada

**Magdalena Cuenca Garcia**

Spain

**Oliver Cumming**

UK

**John Cummings**

UK

**Sharon Cummins**

USA

**Joana Cunha-Cruz**

USA

**Michelle Cunich**

Australia

**Colette Cunningham-Myrie**

Jamaica

**Douglas Curran-Everett**

USA

**Mahomed Dada**

UK

**Ann Dadich**

Australia

**Darren Dahly**

Ireland

**Eleonora Dal Grande**

Australia

**Luigino Dal Maso**

Italy

**Paulo de Tarso Dalcin**

Brazil

**Lance Dalleck**

USA

**Anne Kjersti Daltveit**

Norway

**Elizabeth D'Amico**

USA

**Goodarz Danaei**

USA

**Kelsey Dancause**

Canada

**Lalit Dandona**

India

**Rakhi Dandona**

India

**Elvira D'Andrea**

Italy

**Karen Daniels**

South Africa

**Tom Daniels**

UK

**Andrew Dannenberg**

USA

**Nadia Danon-Hersch**

Switzerland

**Maria Julia Dantur Juri**

Argentina

**Magdy Darwish**

Saudi Arabia

**Ashis Das**

USA

**Jai Das**

Pakistan

**Sudip Datta Banik**

Mexico

**Asli Davas**

Turkey

**Danielle Davidov**

USA

**Alice Davis**

UK

**Stephanie Davis**

USA

**Brenda Davy**

USA

**Mohamed Ali Daw**

Libya

**Halima Dawood**

South Africa

**Anna Dawson**

Australia

**Carolyn Day**

Australia

**Joop de Beer**

Netherlands

**Guy de Bruyn**

USA

**Bart De Clercq**

Belgium

**Nele De Cuyper**

Belgium

**Maria De Iorio**

UK

**Evelyne de Leeuw**

Australia

**Susan De Luca**

USA

**Tim De Maayer**

South Africa

**Judith de Meij**

Netherlands

**Augusto Cesar de Moraes**

Brazil

**Giuseppe De Palma**

Italy

**Laure de Preux**

UK

**Delphine De Smedt**

Belgium

**Anniza De Villiers**

South Africa

**Pol De Vos**

Belgium

**Chiara de Waure**

Italy

**Leonore M. de Wit**

Netherlands

**Kevin Deane**

UK

**James Dearing**

USA

**Zelalem Yilma Debebe**

Ethiopia

**Kora DeBeck**

Canada

**Alex Debrah**

Ghana

**Xavier Debussche**

France

**Eric Dede**

UK

**Martin Dedicoat**

UK

**Kathleen Deering**

Canada

**Abraham Degarege**

Ethiopia

**Olivier Degomme**

Belgium

**Francine Dehue**

Netherlands

**Jocelyn DeJong**

Lebanon

**Rosa del Angel**

Mexico

**Joseph Delaney**

USA

**Louise Delany**

New Zealand

**Monique Delforterie**

Netherlands

**Christine Delgado**

USA

**Tom Deliens**

Belgium

**Hélène Delisle**

Canada

**Thérèse Delvaux**

Benin

**Anahit Demirchyan**

Armenia

**Meaza Demissie**

Ethiopia

**Paddy Dempsey**

Australia

**JM (Lea) den Broeder**

Netherlands

**Chantal den Daas**

Netherlands

**Casper den Heijer**

Netherlands

**Amenu Wesen Denegetu**

Ethiopia

**Rachel Denholm**

France

**Srdjan Denic**

United Arab Emirates

**Lynette Denny**

South Africa

**Simon Denny**

New Zealand

**Licia Denti**

Italy

**Kebede Deribe**

Ethiopia

**Amare Deribew**

Ethiopia

**Hanne Derycke**

Belgium

**Jean-Claude Desenclos**

France

**Pradeep Deshmukh**

India

**Sameer Deshpande**

Canada

**Roger Detels**

USA

**Delan Devakumar**

UK

**Shayesta Dhalla**

Canada

**Nafeesa Dhalwani**

UK

**Ana Angelica Dias**

Brazil

**Esperanza Diaz**

Norway

**Bob Dick**

Australia

**Petra Dickmann**

UK

**Ralph DiClemente**

USA

**Phillippa Diedrichs**

UK

**Marjolein Dieleman**

Netherlands

**Peggye Dilworth-Anderson**

USA

**Christina Dimopoulou**

Germany

**Melody Ding**

Australia

**Joerg Dirmaier**

Germany

**Carlos Disogra**

Argentina

**Maria Diuk-Wasser**

USA

**Mamuka Djibuti**

Georgia

**Bosiljka Djikanovic**

Serbia

**Nga Do**

Viet Nam

**Aram Dobalian**

USA

**Graeme Docherty**

UK

**Stacy Dodd**

USA

**Lawrence Doi**

UK

**Alexander Domnich**

Italy

**Carolin Donath**

Germany

**Lars Donath**

Switzerland

**Lucy Doos**

UK

**Fernanda Dorea**

Canada

**Maria Dorrucci**

Italy

**Svetlana Vladislavovna Doubova**

Mexico

**Sarah Doucette**

Canada

**Flora Douglas**

UK

**Margaret Douglas**

UK

**Michael Doumas**

Greece

**John Doupis**

Greece

**Ian Down**

Australia

**Michael Doyle**

USA

**Ann Dozier**

USA

**Amy Drake**

USA

**Bettina Drake**

USA

**Catherine Draper**

South Africa

**Alex Dregan**

UK

**Robert Dreibelbis**

USA

**Maren Dreier**

Germany

**Clemens Drenowatz**

USA

**Ann Drobnik**

USA

**David Dror**

India

**Francoise Dubois-Arber**

Switzerland

**Katrina DuBose**

USA

**Dean Dudley**

Australia

**Lara Dugas**

USA

**Vesselka Duleva**

Bulgaria

**James Dunbar**

Australia

**Michael Duncan**

UK

**Glen Duncan**

USA

**Ruth Dundas**

UK

**Tinashe Dune**

Australia

**Sally Dunlop**

Australia

**Ana Duran**

Brazil

**Paolo Durando**

Italy

**Sarah Durkin**

Australia

**Daniel Dutton**

Canada

**Laura Dwyer-Lindgren**

USA

**David Dzewaltowski**

USA

**Jaya Earnest**

Australia

**Matthew Eastin**

USA

**John Eastwood**

Australia

**Keith Eastwood**

Australia

**Kristie Ebi**

USA

**Kristina Edvardsson**

Sweden

**Benjamin Edwards**

Australia

**Charlotte Edwardson**

UK

**Frida Eek**

Sweden

**Gudina Egata**

Ethiopia

**Bolaji Egbewale**

Nigeria

**Grace Egeland**

Norway

**Sander Matthijs Eggers**

Netherlands

**Thaddaeus Egondi**

Kenya

**Diane Ehlers**

USA

**Lars Ehlers**

Denmark

**Deborah Ehrenthal**

USA

**Rodney Ehrlich**

South Africa

**Veroni Eichelsheim**

Netherlands

**Rochelle Eime**

Australia

**Michael Eisenberg**

USA

**Orjan Ekblom**

Sweden

**Ibrahim El Bayoumy**

Kuwait

**Awatef El Moussi**

Tunisia

**Theodoros Eleftheriadis**

Greece

**Jessica Elf**

USA

**Kelly Elimian**

UK

**Lorraine Elit**

Canada

**Ari Elliot**

USA

**Susan Elliott**

Canada

**Matthew Ellis**

UK

**Hesham El-Sayed**

Egypt

**Maged El-Setouhy**

Saudi Arabia

**Jon Ivar Elstad**

Norway

**Rune Elvik**

Norway

**J. Mark Elwood**

New Zealand

**Khashaba Eman**

Egypt

**Mustaffa Embong**

Malaysia

**Herb Emery**

Canada

**Ingunn Engebretsen**

Norway

**Sidnei Epelman**

Brazil

**Rajiv Erasmus**

South Africa

**Jennifer Toller Erausquin**

USA

**Berhanu Erko**

Ethiopia

**Julia Ershova**

USA

**Jenni Ervasti**

Finland

**Paul Erwin**

USA

**Veronica Escamilla**

USA

**Jorge Escobedo**

Mexico

**Reuben Escorpizo**

USA

**Emee Estacio**

UK

**Ramon Estruch**

Spain

**Tadele Eticha**

Ethiopia

**Anthony Etyang**

Kenya

**Eva Eurenius**

Sweden

**Charlotte Evans**

UK

**William Evans**

USA

**Sara Evans-Lacko**

UK

**Rebecca Evans-Polce**

USA

**Emma Everson-Hock**

UK

**Ben Ewald**

Australia

**Amy Eyler**

USA

**Emma Eyre**

UK

**Eugene Ezebilo**

Sweden

**Oliver Chukwujekwu Ezechi**

Nigeria

**Nicole Ezendam**

Netherlands

**Fabio Fabbian**

Italy

**Fabrizio Faggiano**

Italy

**Tom Fahey**

Ireland

**Lesley Fairley**

UK

**Victoria Fan**

USA

**Hildegunn Fandrem**

Norway

**Jiming Fang**

Canada

**Mark Faries**

USA

**Andrea Farioli**

Italy

**Muhammad Assad Farooqui**

Singapore

**Matthew Farrelly**

USA

**Karen Farris**

USA

**Konstantinos Farsalinos**

Greece

**Ahmed Fathelrahman**

Saudi Arabia

**Zafar Fatmi**

Pakistan

**Oliver Faude**

Switzerland

**Bukola Fawole**

Nigeria

**Olufunmilayo Fawole**

Nigeria

**Stefano Federici**

Italy

**Bruno Federico**

Italy

**Alan Feingold**

USA

**Lydia Feinstein**

USA

**Berhanu Feleke**

Ethiopia

**Eleonora Feletto**

Australia

**Lambert Felix**

UK

**Muriel Fender**

France

**Cindy Feng**

Canada

**Xiaoqi Feng**

Australia

**Elizabeth Ferdinand**

Barbados

**Ruwan Ferdinando**

Sri Lanka

**Christopher Ferguson**

USA

**Renee Ferguson**

Australia

**Amy Ferketich**

USA

**Rogerio Fermino**

Brazil

**Lorna Fern**

UK

**Romulo Fernandes**

Brazil

**Esteve Fernandez**

Spain

**Maria Luz Fernandez**

USA

**Juan Miguel Fernandez-Alvira**

Spain

**Daniel Fernandez-Berges**

Spain

**Alize Juliette Ferrari**

Australia

**Gerson Luis de Moraes Ferrari**

Brazil

**Marica Ferri**

Portugal

**Kirsten Fiest**

Canada

**Theodosios Filippatos**

Greece

**Filippos Filippidis**

UK

**Gunther Fink**

USA

**Per Fink**

Denmark

**Madelon Finkel**

USA

**Eric Finkelstein**

Singapore

**Jocelyn Finlay**

USA

**Mario Fioravanti**

Italy

**Katherine Fiori**

USA

**Karin Fisher**

Australia

**Eugene Fitzhugh**

USA

**Valerie Flax**

USA

**Jennifer Flegg**

UK

**Lena Fleig**

Canada

**Steffen Flessa**

Germany

**Diego Flichman**

Argentina

**Ellen Flint**

UK

**Jennifer Flood**

USA

**Ewa Florek**

Poland

**Alina Flores**

USA

**George Fodor**

Canada

**Louise Foley**

UK

**Dean Follmann**

USA

**Virginia Fonner**

USA

**Maria João Fonseca**

Portugal

**Lindsay Forbes**

UK

**Allison Ford**

UK

**Brian Ford**

USA

**Eric Ford**

USA

**Jodi Ford**

USA

**Maria Joao Forjaz**

Spain

**James Forrest**

Australia

**Yvonne Forsell**

Sweden

**Alice Forster**

UK

**Emma Foster**

UK

**Carla Fourie**

South Africa

**Sotirios Fouzas**

Greece

**Aaron Fox**

USA

**Ken Fox**

UK

**Elisabetta Franco**

Italy

**Richard Franklin**

Australia

**Eleonor Fransson**

Sweden

**Shannon Frattaroli**

USA

**Rosanne Freak-Poli**

Australia

**John Frean**

South Africa

**Becky Freeman**

Australia

**Paul Freeman**

USA

**David French**

UK

**Atle Fretheim**

Norway

**M. Reuel Friedman**

USA

**Mark Friedman**

USA

**Sharon Friel**

Australia

**Victoria Frye**

USA

**Junfen Fu**

China

**Steven Fu**

USA

**Adrian Fuente**

Australia

**Kayo Fujimoto**

USA

**Yoshiharu Fukuda**

Japan

**Birgit Fullerton**

Germany

**Katie Fullerton**

USA

**Erika Fulmer**

USA

**Gordon Fung**

USA

**Maria-Jose Fuster-RuizdeApodaca**

Spain

**Eszter Füzéki**

Germany

**Márcia G M Alves**

Brazil

**Belinda Gabbe**

Australia

**Sabine Gabrysch**

Germany

**Maria Rosaria Galanti**

Sweden

**Seana Gall**

Australia

**Jane Gallagher**

USA

**Katia Gallegos-Carrillo**

Mexico

**Bruna Galobardes**

UK

**Kristi Gamarel**

USA

**Esteban Gandara**

Canada

**Rashmi Gangamma**

USA

**Marie Gantz**

USA

**Hui Gao**

China

**Yang Gao**

Hong Kong

**Dana Garbarski**

USA

**Lourdes Garcia**

Mexico

**Sandra Garcia**

Colombia

**Antonio García-Hermoso**

Chile

**Benjamin Gardner**

UK

**Kevin Garey**

USA

**Pernilla Garmy**

Sweden

**Paul Garner**

UK

**Fania Ruth Gärtner**

Netherlands

**Mark Garzotto**

USA

**Danijela Gasevic**

Canada

**Tania Gaspar**

Portugal

**Jose M Gatell**

Spain

**Peter Gates**

Australia

**Robin Gauld**

New Zealand

**Anelise Gaya**

Brazil

**Ezra Gayawan**

Nigeria

**Grace Gbotosho**

Nigeria

**Emma Gearon**

Australia

**Klaus Gebel**

Australia

**Alem Gebremariam**

Ethiopia

**Mekdes Gebremariam**

Norway

**Sarah Geiger**

USA

**Aschalew Gelaw**

Ethiopia

**Becky Genberg**

USA

**Joan Gene Badia**

Spain

**Sanne Gerards**

Netherlands

**Dionne Gesink**

Canada

**Anat Gesser-Edelsburg**

Israel

**Meaza Gezu**

Ethiopia

**Huda Gharaibeh**

Jordan

**Akhgar Ghassabian**

USA

**Simone Ghislandi**

Italy

**Philippe Giabbanelli**

Canada

**Peter Giacobbi**

USA

**George Giannakopoulos**

Greece

**Alessandro Giannattasio**

Italy

**Maria Gianni'**

Italy

**Nicola Gianotti**

Italy

**Angelo Giardino**

USA

**Luana Giatti**

Brazil

**Benjamin Gibbs**

USA

**Sigrid Gibson**

UK

**Peter Gichangi**

Kenya

**Angel Gil**

Spain

**Hazel Gilbert**

UK

**Marion Gillen**

USA

**Ardyth Gillespie**

USA

**Conor Gilligan**

Australia

**William Gilmore**

Australia

**Rosa Gini**

Italy

**Gary Ginsberg**

Israel

**Gabriele Giorgi**

Italy

**Paolo Giorgi Rossi**

Italy

**Eshetu Girma**

Ethiopia

**Samuel Girma**

Ethiopia

**Mika Gissler**

Finland

**Massimo Giuliani**

Italy

**Marina Giuliano**

Italy

**Muluken Gizaw**

Ethiopia

**Arjan Gjonca**

UK

**Stanton Glantz**

USA

**Cornelia Glaser**

Germany

**Tavis Glassman**

USA

**Sara Glick**

USA

**Jerome Goddard**

USA

**Irma Godoy**

Brazil

**Marshall Godwin**

Canada

**Naveen Goel**

India

**Shifalika Goenka**

India

**Carlos Gois**

Portugal

**Shira Goldenberg**

USA

**Srinivas Goli**

India

**Gabriela Gomes**

Portugal

**Javier Gomez de Terreros**

Spain

**Diego Gómez-Arbeláez**

Colombia

**Cathy Gong**

Australia

**Consuelo Gonzalez-Suarez**

Philippines

**Sampatha Goonewardena**

Sri Lanka

**Bamini Gopinath**

Australia

**Giuseppe Gorini**

Italy

**George Gotsadze**

Georgia

**Annabelle Gourlay**

UK

**Luis Gracia-Marco**

UK

**Veronica Graham**

Australia

**Michael Grandner**

USA

**Genevieve Grant**

Australia

**Liz Grant**

UK

**Michael Grasso**

USA

**Ben Gray**

New Zealand

**Casey Gray**

Canada

**Cindy Gray**

UK

**Lesley Gray**

New Zealand

**Selena Gray**

UK

**Michael James Green**

UK

**Tim Green**

Canada

**Darren C Greenwood**

UK

**Dario Gregori**

Italy

**Ted Greiner**

Korea, South

**Ursula Griebler**

Austria

**Jessica Grieger**

Australia

**Tyson Grier**

USA

**Amy Griffin**

Australia

**Carley Grimes**

Australia

**Ulrike Grittner**

Germany

**Peter Groenewegen**

Netherlands

**Erik Groessl**

USA

**Wim Grooten**

Sweden

**Angelique Grosser**

Germany

**Ellie Grossman**

USA

**Michael Grover**

USA

**Allison Groves**

USA

**Oliver Gruebner**

USA

**Anne Grundy**

Canada

**Klaus G. Grunert**

Denmark

**Amelia Guadalupe-Grau**

Denmark

**Thomas Guadamuz**

Thailand

**Maria Rosaria Gualano**

Italy

**Giovanni Guaraldi**

Italy

**Leonor Guariguata**

Belgium

**Jonathan Gubbay**

Canada

**Jessica Sophia Gubbels**

Netherlands

**Paulo Guerra**

Brazil

**Gil Guerra-Junior**

Brazil

**Fernando Guerrero-Romero**

Mexico

**Montserrat Guillen**

Spain

**Benjamin Guinhouya**

France

**Unjali Gujral**

USA

**Laszlo Gulacsi**

Hungary

**Gabriel Gulis**

Denmark

**Martin Gulliford**

UK

**Amelia Gulliver**

Australia

**Anil Gumber**

UK

**Valentina Gumenyuk**

USA

**Jin Guo**

China

**Xinbiao Guo**

China

**Hongxiong Guo**

China

**Bhawna Gupta**

India

**Rajeev Gupta**

India

**Sanjeev Gupta**

India

**Shailvi Gupta**

USA

**Mirjana Gurinovic**

Serbia

**Juan Pablo Gutierrez**

Mexico

**Peter Gutierrez**

USA

**Juan Luis Gutierrez-Fisac**

Spain

**Gerda-Maria Haas**

Germany

**Melissa Habel**

USA

**Jessica Haberer**

USA

**Beverley Haddad**

South Africa

**Nyssa Hadgraft**

Australia

**Michiel Haeseker**

Netherlands

**Holly Hagan**

USA

**Lars Hagberg**

Sweden

**Erin Hager**

USA

**Gareth Hagger-Johnson**

UK

**Ali Akbar Haghdoost**

Iran

**Fahimeh Haghighatdoost**

Iran

**Maria Hagstromer**

Sweden

**Judith Hahn**

USA

**Nemat Hajeebhoy**

Viet Nam

**Amal Halder**

Bangladesh

**Danielle Haley**

USA

**Laurence Halimi**

France

**Brian Hall**

China

**Mary Hall**

UK

**Pedro Hallal**

Brazil

**Mats Hallgren**

Sweden

**Randah Hamadeh**

Bahrain

**Mark Hamer**

UK

**Andrew Hamilton**

Australia

**Jan Hamling**

UK

**Anne Hammarström**

Sweden

**Oliver Hämmig**

Switzerland

**Kirsten Hancock**

Australia

**Johanna Hanefeld**

UK

**Sebastien Haneuse**

USA

**Kurt Hanevik**

Norway

**David Hanna**

USA

**Philip Hannaford**

UK

**Germaine Hanquet**

Belgium

**Bjorge Herman Hansen**

Norway

**Claus D. Hansen**

Denmark

**Sonja Hansen**

Germany

**Heidi Hanson**

USA

**Chun Hao**

USA

**Zaeem Haq**

Pakistan

**Sobia Haqqi**

Pakistan

**Sherilee Harper**

Canada

**Jane Harries**

South Africa

**Daniel Harrington**

Canada

**Deirdre Harrington**

UK

**Elizabeth Harris**

Australia

**Jennifer Harris**

USA

**Neil Harris**

Australia

**Patrick Harris**

Australia

**Ingunn Harstad**

Norway

**Christiane Hartog**

Germany

**Stephen Haslett**

New Zealand

**Lamiece Hassan**

UK

**Jameela Hassanali**

Kenya

**Rebecca Hasson**

USA

**Abigail Hatcher**

South Africa

**Susan Hatters Friedman**

New Zealand

**Angelos Hatzakis**

Greece

**Yvonne Hauck**

Australia

**Severin Haug**

Switzerland

**Arie Havelaar**

Netherlands

**Jennifer Havens**

USA

**Stephen Hawes**

USA

**Mary Hawk**

USA

**Kanna Hayashi**

Canada

**Adrian Hayes**

UK

**Alison Hayes**

Australia

**Laura Hayman**

USA

**Ann Haynos**

USA

**Xuesong He**

China

**Benjamin Healey**

New Zealand

**Caroline Heary**

Ireland

**Gregory Heath**

USA

**Nick Heather**

UK

**William Heerman**

USA

**Kristiann Heesch**

Australia

**Jane Heffernan**

Canada

**René Hefti**

Switzerland

**Rebecca Heidkamp**

USA

**Marieke Heijnen**

UK

**Oskari Heikinheimo**

Finland

**Katriina Heikkila**

Finland

**Robert Heimer**

USA

**Katie Heinrich**

USA

**Gilles Hejblum**

France

**Harri Helajärvi**

Finland

**Shaun Helman**

UK

**Stefanie Helmer**

Germany

**Georges Hemstreet**

USA

**Maggie Hendry**

UK

**Belinda Hengel**

Australia

**Ralf Henkel**

South Africa

**Eilis Hennessy**

Ireland

**Emily A. Hennessy**

USA

**Erin Hennessy**

USA

**Devon Hensel**

USA

**Stanley Henshaw**

USA

**Ebiere Herbertson**

Nigeria

**Sonia Hernández-Cordero**

Mexico

**Victoria Hernando**

Spain

**Veronica Herrera**

Chile

**Víctor Herrera**

Colombia

**Emily Herrett**

UK

**Kathryn Hesketh**

UK

**Sonja Hess**

USA

**Lise Hestbaek**

Denmark

**Ingrid Heuch**

Norway

**Barbara Heude**

France

**Gholamreza Heydari**

Iran

**Michael Hiete**

Germany

**Darrel Higa**

USA

**George E. Higgins**

USA

**Vanessa Higgins**

UK

**Peter Higgs**

Australia

**Allan Hill**

UK

**Toni Hilland**

Australia

**Janet Hiller**

Australia

**Danny Hills**

Australia

**Melvyn Hillsdon**

UK

**Erica Hinckson**

New Zealand

**Andrew Hinde**

UK

**Sven Gudmund Hinderaker**

Norway

**Trina Hinkley**

Australia

**Taina Hintsa**

Finland

**Mitsuaki Hirai**

Japan

**Yoichiro Hirakawa**

Australia

**Vasant Hirani**

Australia

**Raquel Hirata**

Brazil

**Caroline Hird**

UK

**Jennifer Hirsch**

USA

**Harriet Hiscock**

Australia

**Ayako Hiyoshi**

Sweden

**Heidi Hjelmeland**

Norway

**Wolfgang Hladik**

Uganda

**Suzanne C Ho**

China

**Erin Hobin**

Canada

**Jason Hockenberry**

USA

**Rebecca Kate Hodder**

Australia

**Allison Hodge**

Australia

**David Hodgins**

Canada

**Paul Hodkinson**

UK

**Janet Hoek**

New Zealand

**Falk Hoffmann**

Germany

**Rasmus Hoffmann**

Netherlands

**Claire Hoffmire**

USA

**Matthew Hogben**

USA

**Hans V Hogerzeil**

Netherlands

**Yara Hokerberg**

Brazil

**Michael Holick**

USA

**Christine Holmberg**

Germany

**Jostein Holmen**

Norway

**Bev J Holmes**

Canada

**Bjørn E. Holstein**

Denmark

**Martin Holt**

Australia

**Wiliam Holzemer**

USA

**Akira Homma**

Brazil

**Yasushi Honda**

Japan

**David Hondula**

USA

**Chang Hyung Hong**

Korea, South

**Jun Sung Hong**

USA

**Paula Hooper**

Australia

**Monjurul Hoque**

South Africa

**Tanya Horacek**

USA

**Samar Hore**

Bangladesh

**Susan Horton**

Canada

**H Dean Hosgood**

USA

**Akiko Hosler**

USA

**Travis Salway Hottes**

Canada

**Stephen Houghton**

Australia

**Emma Howarth**

UK

**Philippa Howden-Chapman**

New Zealand

**Rosalind Howes**

UK

**Harald Hrubos-Strøm**

Norway

**Howard Hu**

USA

**Guoqing Hu**

China

**Mi Hu**

China

**Yun Hu**

China

**Xiaomei Hu**

China

**Jenna Hua**

USA

**De-Sheng Huang**

China

**Wan-Ting Huang**

Taiwan

**Yong Huang**

China

**Zhihuan Huang**

USA

**Anne Hublet**

Belgium

**Fauzia Akhter Huda**

Bangladesh

**Ian Hughes**

Australia

**Nathalie Huguet**

USA

**Jimi Huh**

USA

**Stanley Hui**

Hong Kong

**Chifa Hung**

Taiwan

**Anna Christina Hunt**

UK

**Ruth Hunter**

UK

**John Hurdle**

USA

**Fintan Hurley**

UK

**Rafat Hussain**

Australia

**Tahziba Hussain**

India

**Lieven Huybregts**

Belgium

**Emily Hyle**

USA

**Alex Iantaffi**

USA

**Hyacinth Ichoku**

Nigeria

**Emmy Igonya**

Kenya

**S. Odunayo Ikuerowo**

Nigeria

**Zubairu Iliyasu**

Nigeria

**Samantha Illangasekare**

USA

**Hironori Imano**

Japan

**Fumio Imazeki**

Japan

**loredana Ingrosso**

Italy

**Shigeru Inoue**

Japan

**Ryusuke Inoue**

Japan

**Tabassum Insaf**

USA

**Michael Iroezindu**

Nigeria

**Brandon Irwin**

USA

**Deus Ishengoma**

Tanzania

**Nobukazu Ishizaka**

Japan

**Mohammed F Islam**

Australia

**Md Rafiqul Islam**

Australia

**Md Robiul Islam**

Australia

**Md. Sirajul Islam**

Bangladesh

**Chimaraoke Izugbara**

Kenya

**Alun Jackson**

Australia

**Charlotte Jackson**

UK

**Cristina Jacob**

Brazil

**Tazeen Jafar**

Singapore

**Jagnoor Jagnoor**

Australia

**Albrecht Jahn**

Germany

**Sara Jahnke**

USA

**Juliana Jalaludin**

Malaysia

**Mohamed Jalloh**

Senegal

**Ahmed Jamal**

USA

**Katherine James**

USA

**Peter James**

USA

**Frances Jamieson**

Canada

**Mohamed Janabi**

Tanzania

**Jonine Jancey**

Australia

**Astrid Jander**

Netherlands

**Hak Chul Jang**

Korea, South

**Katharina Janke**

UK

**Urban Janlert**

Sweden

**Christian Janssen**

Germany

**Imke Janssen**

USA

**Pande Putu Januraga**

Indonesia

**Edward Janus**

Australia

**Prawit Janwantanakul**

Thailand

**Bonnie Janzen**

Canada

**Jennifer Jao**

USA

**Peter Jarcuska**

Slovakia

**Domantas Jasilionis**

Germany

**Barbara Jauregui**

USA

**Marjan Javanbakht**

USA

**Radhakrishnan Jayakrishnan**

India

**Upali W. Jayasinghe**

Australia

**Rohan Jayasuriya**

Australia

**Ranil Jayawardena**

Sri Lanka

**Seyed Mohammad Jazayeri**

Iran

**Barbara Jefferis**

UK

**Alison Jeffery**

UK

**Jennifer Margaret Jelsma**

South Africa

**Dawn Jenkin**

UK

**Helen Jenkins**

USA

**Chris Jensen**

Denmark

**Man Jung Jeon**

Korea, South

**Charlotte Jeppesen**

Denmark

**Gyorgy Jermendy**

Hungary

**Yijun Jia**

China

**Silvia Jimenez-Jorge**

Spain

**Kerri Johannson**

Canada

**Anders Johansson**

UK

**Roar Johnsen**

Norway

**Blair Johnson**

USA

**Newell Johnson**

Australia

**Richard Johnson**

USA

**Timothy Johnson**

USA

**Vicki Johnson-Lawrence**

USA

**Carol Johnston**

USA

**Lisa Johnston**

USA

**Outi Jolanki**

Finland

**Karlijn J Joling**

Netherlands

**Andy Jones**

UK

**Charlotte Jones**

Canada

**Andrew Jones**

USA

**Sarah Jones**

UK

**Kelly Jones**

Australia

**Lisa Jones**

UK

**Natalia Jones**

UK

**Michelle Jongenelis**

Australia

**Stefan Hrafn Jonsson**

Iceland

**Marie Birk Jorgensen**

Denmark

**Louisa Jorm**

Australia

**Kim Jose**

Australia

**Nitin Joseph**

India

**Noyal Joseph**

India

**Rodney Joseph**

USA

**Malin Josephson**

Sweden

**Roshni Joshi**

UK

**Rong-Chang Jou**

Taiwan

**Sol Juárez**

Sweden

**Jenni Judd**

Australia

**Niina Junttila**

Finland

**Markus Juonala**

Finland

**Anamarija Jurcev-Savicevic**

Croatia

**Abdunoor Mulokozi Kabanywanyi**

Tanzania

**Zubair Kabir**

Ireland

**Sushil Kabra**

India

**Lydia Kaduka**

Kenya

**Michael Kaess**

Germany

**Juliana Kagura**

South Africa

**Juliana Kain**

Chile

**Lucia Kaiser**

USA

**Raj Kalaria**

UK

**Dorota Kaleta**

Poland

**Håkan Källmén**

Sweden

**Lucie Kalousova**

USA

**Sebastian Kalwij**

UK

**Joan Kalyango**

Uganda

**Mphatso Kamndaya**

Malawi

**Carlijn Kamphuis**

Netherlands

**Bavesh Davandra Kana**

South Africa

**Dianmin Kang**

China

**Yoon-Jung Kang**

Australia

**Umamaheswari Kannan**

India

**Teshome Gebre Kanno**

Ethiopia

**Syeda Kanwal**

Pakistan

**Nilamadhab Kar**

UK

**Charles Amnon Sunday Karamagi**

Uganda

**Norimah Karim**

Malaysia

**Arun Karlamangla**

USA

**Ioannis Karydis**

Greece

**Adetayo Kasim**

UK

**Milica Katic**

Croatia

**Maram Katoue**

Kuwait

**Ingrid Katz**

USA

**Sherri Katz**

Canada

**Jay Kaufman**

Canada

**Prabhdeep Kaur**

India

**Noy Kay**

USA

**Vanessa Kay**

UK

**Gurhan Kayihan**

UK

**Bengt Kayser**

Switzerland

**Aadil Kazi**

UK

**Sarah Keadle**

USA

**Benjamin Kearns**

UK

**Lisa Keay**

Australia

**Getahun Kebede Beyera**

Ethiopia

**Richard Keegan**

Australia

**Michelle Kegler**

USA

**Hunyinbo Kehinde Isaac**

Nigeria

**Margaret Kelaher**

Australia

**Helen Keleher**

Australia

**Roya Kelishadi**

Iran

**John Kellett**

Ireland

**Bridget Kelly**

Australia

**Colette Kelly**

Ireland

**Louise Kelly**

USA

**Paul Kelly**

UK

**Shona Kelly**

UK

**Louise Kelly-Hope**

UK

**Han CG Kemper**

Netherlands

**Friederike Kendel**

Germany

**Andre Pascal Kengne**

South Africa

**Norelee Kennedy**

Ireland

**Sara Kennedy**

USA

**Deanna Kepka**

USA

**Brent Kerger**

USA

**Janko Kersnik**

Slovenia

**Ammar H Keshteli**

Canada

**Joanna Kesten**

UK

**Saman Khalesi**

Australia

**Naila Khalil**

USA

**Amina Khambalia**

Australia

**Uzma Khan**

Pakistan

**Yasmin Khan**

Canada

**Vishnu Khanal**

Nepal

**Young-Ho Khang**

Korea, South

**Issam Khatib**

Papua New Guinea

**Olga Khavjou**

USA

**Hossein Khosravi Boroujeni**

Australia

**Haider A. Khwaja**

USA

**Meriem Khyatti**

Morocco

**Nima Kianoush**

Australia

**Mulugeta Kibret**

Ethiopia

**Judi Kidger**

UK

**Rachel Kidman**

USA

**Michele Kiely**

USA

**Kim Kiely**

Australia

**Mairead Kiely**

Ireland

**Juliet Kiguli**

Uganda

**Reinhold Kilian**

Germany

**Jong Yeol Kim**

Korea, South

**Sun Kim**

USA

**Susan Kim**

Australia

**Elizabeth Kimani-Murage**

Kenya

**Brian King**

USA

**Carina King**

UK

**Daniel King**

Australia

**Malcolm King**

Canada

**Matthew King**

USA

**Karl Kingsley**

USA

**Stuart Kinner**

Australia

**Sanjay Kinra**

UK

**Janni Kinsler**

USA

**John Kinsman**

Sweden

**Elizabeth Kiracho**

Uganda

**James Bowes Kirkbride**

UK

**Irma Kirtadze**

Georgia

**Svein R. Kjosavik**

Norway

**Heidi Klakk**

Denmark

**Alexandra Kleiman**

Germany

**Evan Kleiman**

USA

**Jakob Klein**

Austria

**Zalika Klemenc-Ketis**

Slovenia

**Knut-Inge Klepp**

Norway

**Stephanie Knaak**

Canada

**Thomas Kneib**

Germany

**Emily Knox**

UK

**David Knox**

USA

**Leslie Kobayashi**

USA

**Mallory Koenings**

USA

**Steven Koester**

USA

**Gavin Koh**

Netherlands

**Gerjo Kok**

Netherlands

**Lauri Kokkinen**

Finland

**Sami Kokko**

Finland

**Tracy Kolbe-Alexander**

Australia

**Ourania Kolokotroni**

Cyprus

**Naoki Kondo**

Japan

**Norella Kong**

Malaysia

**Evangelos Kontopantelis**

UK

**Winston Koo**

USA

**Mohammad Javad Koohsari**

Australia

**Eran Kopel**

Israel

**Rachel Kornfield**

USA

**Karolina Kósa**

Hungary

**Jussi Kosola**

Finland

**Michael Kostapanos**

Greece

**Konstantinos Kostikas**

Greece

**Catherine Kothari**

USA

**Emily Kothe**

Australia

**Taiwo Kotila**

Nigeria

**Efstathios Koulourides**

Greece

**Anne Kouvonen**

Finland

**Fiona Kouyoumdjian**

Canada

**Gabor Kovacs**

Hungary

**John Koval**

Canada

**Naoko Kozuki**

USA

**Vivica Kraak**

USA

**M. Kaye Kramer**

USA

**Ines Krass**

Australia

**Julia Kravchenko**

USA

**Katrina Kretsinger**

USA

**Gary Krieger**

USA

**Edward Krisiunas**

USA

**Abhay Kudale**

India

**Sharon Kuhlmann**

Sweden

**Louise Kuhn**

USA

**Nicole Kuiper**

USA

**Mirte Kuipers**

Netherlands

**Ivana Kulhánová**

Netherlands

**David Kulick**

USA

**Martin Kulldorff**

USA

**Jenni Kulmala**

Finland

**Shuzo Kumagai**

Japan

**Pratap Kumar**

Kenya

**Santosh Kumar**

USA

**Supriya Kumar**

USA

**Abera Kumie**

Ethiopia

**Anton Kunst**

Netherlands

**Caroline Kuo**

USA

**Pablo Kuri-Morales**

Mexico

**Terhi Kurko**

Finland

**Kalle Kurppa**

Finland

**Hannamaria Kuusio**

Finland

**Keisuke Kuwahara**

Japan

**Japheth Kwiringira**

Uganda

**Man Ki Kwok**

Hong Kong

**Jeff Kwong**

Canada

**Daniele La Rosa**

Italy

**Mikko Laaksonen**

Finland

**Ronald Labonte**

Canada

**Lara LaCaille**

USA

**Ugo Lachapelle**

Canada

**Francine Laden**

USA

**Joel Ladner**

France

**Maria João Lagoa**

Portugal

**Nathalie Lahoud**

Lebanon

**Jouni Lahti**

Finland

**Wen-Ter Lai**

Taiwan

**Robert Laird**

USA

**Amelia Lake**

UK

**Jeroen Lakerveld**

Netherlands

**Yihunie L Lakew**

Ethiopia

**Lawrence T. Lam**

Hong Kong

**Tina Lam**

Australia

**Karen Lamb**

Australia

**Estelle Lambert**

South Africa

**Dorian Lamis**

USA

**Steven Lamm**

USA

**Cristina Lammers**

USA

**Anthony Daniel LaMontagne**

Australia

**Sebastien Lamy**

France

**Timothy Landers**

USA

**Ellen Melbye Langballe**

Norway

**John Lange**

USA

**Rebecca Langford**

UK

**Tessa Langley**

UK

**kamran Lankarani**

Iran

**Malinee Laopaiboon**

Thailand

**Nancy LaPelle**

USA

**Linda Larcombe**

Canada

**Francesca Larese Filon**

Italy

**Michel Lariviere**

Canada

**Celine Larkin**

Ireland

**Sarah Larkins**

Australia

**Amparo Larrauri**

Spain

**David Larsen**

USA

**Torill Larsen**

Norway

**Bruce Larson**

USA

**Celia Larson**

USA

**Elysia Larson**

USA

**Heidi Larson**

UK

**Mary Jo Larson**

USA

**Timothy Lash**

USA

**Melissa Nelson Laska**

USA

**Romy Lauche**

Germany

**Arto Laukkanen**

Finland

**Henrik Hein Lauridsen**

Denmark

**Anthony Laverty**

UK

**Carl Lavie**

USA

**Francine Lavoie**

Canada

**CK Law**

Hong Kong

**Sharon Lawn**

Australia

**Taiwo Lawoyin**

Nigeria

**Mark Lawrence**

Australia

**Rachel Laws**

Australia

**Giacomo Lazzeri**

Italy

**Marzia Lazzerini**

Italy

**Tuan Le**

USA

**Jacques Le Houezec**

France

**Stanzi le Roux**

South Africa

**Vania Aparecida Leandro-Merhi**

Brazil

**Cynthia A. Leaver**

USA

**Charlotte Leboeuf-Yde**

Denmark

**Lilian Lechner**

Netherlands

**Glenn Leckie**

USA

**Annette Leclerc**

France

**Albert Lee**

Hong Kong

**Andrew Chee Keng Lee**

UK

**Byung-Kook Lee**

Korea, South

**Chioun Lee**

USA

**Mingshinn Lee**

Taiwan

**Marilyn Lee**

Canada

**Paul Lee**

Hong Kong

**Wen-Chung Lee**

Taiwan

**Yunhwan Lee**

Korea, South

**Young-Mi Lee**

USA

**Shelley Lees**

UK

**Luc Leger**

Canada

**Mengistu Legesse**

Ethiopia

**Sandra Leggat**

Australia

**Jonathon Leider**

USA

**Marie Leiner**

USA

**Mall Leinsalu**

Sweden

**Margus Lember**

Estonia

**Laetitia Lempereur**

Belgium

**Kelly L'Engle**

USA

**Ryan Lennon**

USA

**Michela Lenzi**

Italy

**David Orlando Leon**

Cuba

**Katherine Lepani**

Australia

**Aurelia Lepine**

UK

**Felice Le-Scherban**

USA

**Catherine Lesko**

USA

**Paola Letona**

Guatemala

**Jessica Leung**

USA

**WM Raymond Leung**

Hong Kong

**Kate Levin**

UK

**Len Levy**

UK

**Daniel Lewis**

UK

**Sophie Lewis**

Australia

**Joel Lexchin**

Canada

**Els Leye**

Belgium

**Jianhua Li**

China

**Kin-Kit Li**

Hong Kong

**Yanbing Li**

China

**Haochu Li**

China

**Kaigang Li**

USA

**Jian Li**

Germany

**Ying Li**

China

**Jialiang Li**

Singapore

**Wenjun Li**

USA

**Hao Liang**

China

**Yuan Liang**

China

**Ying Liang**

China

**Yilan Liao**

China

**Su su Liao**

China

**Massimo Libra**

Italy

**Arve Lie**

Norway

**Stein Atle Lie**

Norway

**Lars Lien**

Norway

**Troels Lillebaek**

Denmark

**Rebbecca Lilley**

New Zealand

**Chih-Lung Lin**

Taiwan

**Jin-Ding Lin**

Taiwan

**Shih-Wen Lin**

USA

**Pao-Hwa Lin**

USA

**Vivian Lin**

Australia

**Melissa Lindeman**

Australia

**Marie Lindkvist**

Sweden

**Bernt Lindtjorn**

Norway

**Kristina Lindvall**

Sweden

**Alissa Link**

USA

**Tsan-Hon Liou**

Taiwan

**Isaac Lipkus**

USA

**Katherine Lippel**

Canada

**Martin Lipsky**

USA

**Anne Liu**

USA

**Kawika Liu**

USA

**Yan Liu**

China

**Ruiling Liu**

USA

**Xiaodong Liu**

USA

**Jennifer Livingston**

USA

**Lisa Lix**

Canada

**Margarida Liz Martins**

Portugal

**Therese Ljungquist**

Sweden

**Francisco Jesus Llorente-Cantarero**

Spain

**Scott Lloyd**

UK

**Siu Hing Lo**

UK

**Serigne Lo**

Australia

**Wilson Lo**

Switzerland

**Stephen Locarnini**

Australia

**Sara Lodi**

USA

**Adrian Loerbroks**

Germany

**Marie Löf**

Sweden

**Christopher A. Loffredo**

USA

**Maria Lohan**

UK

**Kiran Loi**

UK

**Lurdes Lomba**

Portugal

**Carl Lombard**

South Africa

**Richard Long**

Canada

**Nicola Longo**

USA

**Thingnganing Longvah**

India

**Pier Luigi Lopalco**

Sweden

**Luís Lopes**

Portugal

**Marcia Edilaine Lopes Consolaro**

Brazil

**Nancy Lopez**

USA

**Ana Lopez-de-Andres**

Spain

**Theo Lorenc**

UK

**Olabisi Loto**

Nigeria

**Margaret Loughnan**

Australia

**Barbara Lourenco**

Brazil

**Karolina Louvanto**

Finland

**Quinette Louw**

South Africa

**Penny Love**

Australia

**Jesper Löve**

Sweden

**Marian Loveday**

South Africa

**John Lowe**

Australia

**David Lubans**

Australia

**Sean Lucan**

USA

**Stanley Luchters**

Australia

**Sara Luckhaupt**

USA

**Anne Ludbrook**

UK

**Alessandra Lugo**

Italy

**Jeremy Luk**

USA

**Amy Luke**

USA

**Joanne Luke**

Australia

**Deus Lukoye**

Uganda

**Pisake Lumbiganon**

Thailand

**Thorsten Lunau**

Germany

**Ingeborg Lund**

Norway

**Rikke Lund**

Denmark

**Patric Lundberg**

Sweden

**Jeffrey C Lunnen**

USA

**Nicole Lurie**

USA

**Katherine Lust**

USA

**Jun Lv**

China

**Brigid Lynch**

Australia

**Fiona Lynn**

UK

**Anthony Lyons**

Australia

**Elizabeth Lyons**

USA

**Pappachan M Joseph**

UK

**Guansheng Ma**

China

**Ping Ma**

USA

**Richard Ma**

UK

**Wei Ma**

China

**Gary Maartens**

South Africa

**Ruth Mabry**

Oman

**Georgie MacArthur**

UK

**Laura MacCalman**

UK

**Emily MacDonald**

Norway

**Sara Macdonald**

UK

**Aristides Machado-Rodrigues**

Portugal

**Shingai Machingaidze**

South Africa

**Natasha Mack**

USA

**Martin Mackey**

Australia

**Barbara MacKinnon**

Canada

**Kelly Mackintosh**

UK

**Ellen Maclachlan**

USA

**Alice MacLean**

UK

**Martha Macleod**

Canada

**Freya MacMillan**

Australia

**Catherine MacPhail**

Australia

**Alison Macpherson**

Canada

**Michelle Macy**

USA

**Barbara Madaj**

UK

**Mohsen Maddah**

Iran

**Jay Maddock**

USA

**Raglan Maddox**

Australia

**Alberto Madeiro**

Brazil

**Sphiwe Madiba**

South Africa

**Ann Madsen**

USA

**Ida Madsen**

Denmark

**Lea Maes**

Belgium

**Anthea Magarey**

Australia

**Matthew Magee**

USA

**Stefania Maggi**

Canada

**Nicola Magnavita**

Italy

**Anne Magnus**

Australia

**Roger Magnusson**

Australia

**Linda Magnusson Hanson**

Sweden

**Tanmay Mahapatra**

USA

**Rajendra Maharaj**

South Africa

**Rohan Maharaj**

Trinidad and Tobago

**Carol Maher**

Australia

**Dermot Maher**

UK

**Mohamed Mahfouz**

Saudi Arabia

**Martin Mahoney**

USA

**Namaitijiang Maimaiti**

Malaysia

**Slawomir Majewski**

Poland

**Shannon Majowicz**

Canada

**Donna Mak**

Australia

**Tomi Mäkinen**

Finland

**Fredrick Makumbi**

Uganda

**Reza Malekzadeh**

Iran

**Kenneth Maleta**

Malawi

**Rahul Malhotra**

Singapore

**Ouattara Mamadou**

Cote d'Ivoire

**Suzanne Maman**

USA

**George Mammen**

Canada

**Alessia Mammone**

Italy

**Hadii Mamudu**

USA

**James Mancuso**

USA

**Samuel Manda**

South Africa

**Amee Manges**

Canada

**Punam Mangtani**

UK

**Jonathan Mann**

Israel

**Michael Mann**

USA

**Leticia Mansur**

Brazil

**David Mant**

UK

**Eleni Mantzari**

UK

**Ann Manzardo**

USA

**Karen Manzo**

USA

**Guangyun Mao**

China

**Alan March**

Australia

**Afrodita Marcu**

UK

**Dilshad Marikar**

UK

**Staffan Marild**

Sweden

**Fatima Marinho**

Brazil

**Adelais Markaki**

Greece

**Francis Markham**

Australia

**Alayne Markland**

USA

**Suzanne Markoe Hayes**

USA

**Laura Marlow**

UK

**Adilson Marques**

Portugal

**Elisa Marques**

Portugal

**Elaine Marqueze**

Brazil

**Elliot Marseille**

USA

**Samantha Marsh**

New Zealand

**Brandon Marshall**

Canada

**LaTisha Marshall**

USA

**Roger Marshall**

New Zealand

**Louise Marston**

UK

**Carmel Martin**

Ireland

**Lisa Martin**

Australia

**Mary Martinasek**

USA

**Jennifer Martin-Biggers**

USA

**Domenico Martinelli**

Italy

**Marianna Martinelli**

Italy

**Maria Martinez**

Brazil

**Priscilla Martinez**

USA

**Cristina Martínez**

Spain

**Judith Martin**-**Fernandez**

France

**Ana Paula Martins**

Brazil

**Chantal Martin-Soelch**

Switzerland

**Ma.Luisa Marvan**

Mexico

**Werner Marx**

Germany

**Joyce Rose Masalu**

Tanzania

**Melkiory Masatu**

Tanzania

**Robert Mash**

South Africa

**Saidur Mashreky**

Bangladesh

**Phil Mason**

UK

**Moses Massaquoi**

Liberia

**Peter Massey**

Australia

**Ruth Masterson Creber**

USA

**Giuseppe Mastrangelo**

Italy

**Stefan Matecki**

France

**Colin Mathers**

Switzerland

**Catriona Matheson**

UK

**Anitha Mathew**

USA

**Catherine Mathews**

South Africa

**Charu Mathur**

USA

**Prashant Mathur**

India

**Joseph Matovu**

Uganda

**Lloyd Matowe**

Liberia

**Konstadinos Mattas**

Greece

**Alberto Matteelli**

Italy

**Giorgio Mattei**

Italy

**Charles Matthews**

USA

**Catherine Maulsby**

USA

**Taro Mawatari**

Japan

**Roy William Mayega**

Uganda

**Christopher Maylahn**

USA

**Bongani Mawethu Mayosi**

South Africa

**Darren Mays**

USA

**Rosie Mayston**

UK

**Mayfong Mayxay**

Laos

**Sigal Mazor**

Israel

**Salig Ram Mazta**

India

**Catherine Mbagaya**

Kenya

**Blessing Mberu**

Kenya

**Eta Ngole Mbong**

Cameroon

**Anthony Mbonye**

Uganda

**Colleen McBride**

USA

**Sara McCafferty**

UK

**Elaine McCarthy**

Ireland

**Heather McCauley**

USA

**Jenna McCauley**

USA

**Gary McClelland**

USA

**Jenny McCloskey**

Australia

**Judith McCool**

New Zealand

**Catriona McDaid**

UK

**Robyn McDermott**

Australia

**Sarah McDonald**

Canada

**Scott McDonald**

Malaysia

**Suzanne McDonough**

UK

**Colin McFarlane**

UK

**Stephen McGarvey**

USA

**Kevin McGeechan**

Australia

**Emily McGibbon**

USA

**Keith McInnes**

USA

**William McKelvie**

Pakistan

**Lyle McKinnon**

South Africa

**C. Jason McKnight**

USA

**Rachael McLean**

New Zealand

**Philip McLoone**

UK

**Brian J McMahon**

USA

**Brian McMillan**

UK

**Juliet McMullin**

USA

**Geraldine McNeill**

UK

**Hayden McRobbie**

UK

**Erin Mead**

USA

**Nathanael Meckes**

USA

**Nicolas Meda**

Burkina Faso

**Araya Abrha Medhanyie**

Ethiopia

**Catalina Medina**

Mexico

**Cristian Meghea**

USA

**Anja Mehnert**

Germany

**Zhang Meilin**

China

**Jessie Meis**

Netherlands

**Wubegzier Mekonnen**

Ethiopia

**Eivind Meland**

Norway

**Alessia Melegaro**

Italy

**Jaymie Meliker**

USA

**René J.F. Melis**

Netherlands

**Fabio Meloni**

Italy

**Craig Melville**

UK

**Glenn Melvin**

Australia

**Marisa Mena**

Spain

**Karla Lorena Mendonça**

Brazil

**Sergio Meneses Navarro**

Mexico

**Ritesh G Menezes**

Saudi Arabia

**Valdenice Menezes**

Brazil

**Mekoya Mengistu**

Ethiopia

**Gwenn Menvielle**

France

**Nicolas Menzies**

USA

**Catherine Mercer**

UK

**Liesbeth Mercken**

Netherlands

**Suzanne Meredith**

UK

**Kim Meredith-Jones**

New Zealand

**Martin Meremikwu**

Nigeria

**Dafna Merom**

Australia

**Michael Merten**

USA

**Arthur Mesas**

Spain

**Nebiyu Mesfin**

Ethiopia

**Giuseppe Alessio Messano**

Italy

**Lynne Messer**

USA

**Brad Metcalf**

UK

**Fraukje Mevissen**

Netherlands

**Jaimie Meyer**

USA

**Sharmila Mhatre**

Canada

**James Michaelson**

USA

**Dominique Michaud**

USA

**Susan Michie**

UK

**Lisa Micklesfield**

South Africa

**Rossella Miglio**

Italy

**Aleksandra Mihailovic**

USA

**Katja Mikhailovich**

Australia

**Rafael T Mikolajczyk**

Germany

**José C. Millán-Calenti**

Spain

**Lynne Millar**

Australia

**Seán R. Millar**

Ireland

**Caroline Louise Miller**

Australia

**Emma Miller**

Australia

**Yvette Miller**

Australia

**Brennen Mills**

Australia

**Allison Milner**

Australia

**Karen Milton**

Australia

**Abul Milton**

Australia

**Mia Minen**

USA

**Krista Lynn Minnotte**

USA

**Stephen James Minton**

Ireland

**Barbara Mintzes**

Canada

**Estrella Miqueleiz**

Spain

**Andrew Mirelman**

USA

**Brian Mishara**

Canada

**Josef Mitas**

Czech Republic

**Jonathan Mitchell**

USA

**Jason Mitchell**

USA

**Marc Mitchell**

Canada

**Sophie Mitra**

USA

**Georgia Mitsi**

USA

**Maurice B Mittelmark**

Norway

**Kyoko Miura**

Australia

**Elia John Mmbaga**

Tanzania

**Ana Olga Mocumbi**

Mozambique

**Pietro Amedeo Modesti**

Italy

**Cameron Moffatt**

Australia

**Feleke Moges**

Ethiopia

**Yasmin Mohamed**

Australia

**Asghar Mohammadpoorasl**

Iran

**Zalilah Mohd Shariff**

Malaysia

**Mohammadreza Mohebbi**

Austria

**Katia Mohindra**

Canada

**Rahim Moineddin**

Canada

**Anu Molarius**

Sweden

**Nicolas Molinari**

France

**Diego Moliner-Urdiales**

Spain

**Liesbeth Mollema**

Netherlands

**Lluis Mont**

Spain

**Anthony Montgomery**

Greece

**Jennifer Moodley**

South Africa

**Ramal Moonesinghe**

USA

**Alison Moore**

USA

**Catrin Moore**

UK

**David Moore**

Canada

**Gabriel Moore**

Australia

**Graham Moore**

UK

**Vasee Moorthy**

Switzerland

**Zohar Mor**

Israel

**Marco Morabito**

Italy

**Sudhakar Morankar**

Ethiopia

**Carla Moreira**

Portugal

**Pedro Moreira**

Portugal

**Sérgio Moreira**

Brazil

**Angelo Moretto**

Italy

**Frederick Morfaw**

UK

**Ian Morgan**

Australia

**Charles Morgan**

USA

**Amani Thomas Mori**

Tanzania

**Yuko Morikawa**

Japan

**Donald Morisky**

USA

**Michela Jane Angela Morleo**

UK

**Mohammad ali Morowatisharifabad**

Iran

**Nancy Morris**

USA

**David Morrison**

USA

**Rory Morrison**

UK

**Dianne Morrison-Beedy**

USA

**Seyed Mohammad Javad Mortazavi**

Iran

**Kevin Mortimer**

UK

**Mosa Moshabela**

South Africa

**Hanns Moshammer**

Austria

**Anne Mosnier**

France

**Harry Moultrie**

South Africa

**Sandra Mounier-Jack**

UK

**Fiona Mowbray**

UK

**Foong Ming Moy**

Malaysia

**Cheryl Moyer**

USA

**Sabrina John Moyo**

Norway

**Elias Mpofu**

Australia

**Filbert Mpogoro**

Tanzania

**Stephen Mshana**

Tanzania

**Peter Muennig**

USA

**Nazeem Muhajarine**

Canada

**Ashar Muhammad Malik**

Pakistan

**Wilson Winstons Muhwezi**

Uganda

**Oscar J. Mujica**

USA

**Agurtzane Mujika**

Spain

**Andrew Mujugira**

Uganda

**Hiroshi Mukae**

Japan

**Arnab Mukherjea**

USA

**Katherine Muldoon**

Canada

**Kate Mulgrew**

Australia

**Barbara Mullan**

Australia

**Carsten Müller**

Germany

**Afework Mulugeta**

Ethiopia

**Joyce Mumah**

Kenya

**Mutale Mumba**

Zimbabwe

**Diego Munguia-Izquierdo**

Spain

**Tiago Munhoz**

Brazil

**Fehmidah Munir**

UK

**Claudia Munoz-Zanzi**

USA

**Adrianna Murphy**

UK

**Alan Murray**

USA

**Megan Murray**

USA

**Nandita Murukutla**

India

**Moji Musa**

South Africa

**Beate Muschalla**

Germany

**Stella Muthuri**

Canada

**Stella Muthuri**

UK

**Raya Muttarak**

Austria

**Twaha Mutyaba**

Uganda

**Adamson Muula**

Malawi

**Conrad Muzoora**

Uganda

**Mercy Mvundura**

USA

**Martha Mwangome**

Kenya

**Matilu Mwau**

Kenya

**Michael Myers**

USA

**Arne Kristian Myhre**

Norway

**Puja Myles**

UK

**Karen Myllynen Webb**

Zimbabwe

**Julie Mytton**

UK

**Paulo Nadanovsky**

Brazil

**Tom Nadarzynski**

UK

**Azadeh Nadjarzadeh**

Iran

**Robert Naeije**

Belgium

**Jason Nagata**

USA

**Csilla Nagy**

Hungary

**Eliana Aguiar Petri Nahas**

Brazil

**Aliya Naheed**

Bangladesh

**Suchithra Naish**

Australia

**Mehdi Najafzadeh**

USA

**Georgina Nakafero**

UK

**Mieko Nakamura**

Japan

**Sarah Nakubulwa**

Uganda

**Solange Nappo**

Brazil

**Steven Narod**

Canada

**Eduardo Nascimento**

USA

**Denis Nash**

USA

**Mercy Nassali**

Bangladesh

**Rose Nathan**

Tanzania

**Javaid Nauman**

Norway

**Ana Navas-Acien**

USA

**Lifoter Navti**

Cameroon

**Patti-Jean Naylor**

Canada

**lyly Nazemi**

Iran

**Martial Ndeffo Mbah**

USA

**Eric Nehl**

USA

**Torsten Neilands**

USA

**Noele Nelson**

USA

**Bjarne Nes**

Norway

**Marion Nestle**

USA

**Lindsay Nettlefold**

Canada

**Yael Netz**

Israel

**Maike Neuhaus**

Australia

**Maria Neumann**

Germany

**Alan Nevill**

UK

**Stephen Neville**

New Zealand

**Jin Rou New**

Singapore

**Bruce Newbold**

Canada

**Susan Newcomer**

USA

**Sophie Newitt**

UK

**Lareen Newman**

Australia

**Peter A Newman**

Canada

**Robert Newton**

USA

**Chiu-Wan Ng**

Malaysia

**Shu Wen Ng**

USA

**Linda Ng Fat**

UK

**Matilda Ngarina**

Tanzania

**Dinh Ngocsy**

Viet Nam

**Mimmie Claudine Ngum Chi Watts**

Australia

**Toai Phuong Nguyen**

Viet Nam

**Chunping Ni**

China

**James Nicholls**

UK

**Gordon Nichols**

Sweden

**Gordon Nichols**

UK

**Theresa Nicklas**

USA

**Loredana Nicoletti**

Italy

**Thomas Niederkrotenthaler**

Austria

**Isabelle Niedhammer**

France

**Anni Brit Sternhagen Nielsen**

Denmark

**Albert Nienhaus**

Germany

**Vera Nierkens**

Netherlands

**Pythia Nieuwkerk**

Netherlands

**Bahareh Nikooyeh**

Iran

**Per Nilsen**

Sweden

**Kerstin Nilsson**

Sweden

**Feng Ning**

Finland

**Nobuo Nishi**

Japan

**Roberto Nisini**

Italy

**Dorothea Nitsch**

UK

**Patricia Njuguna**

Kenya

**Peter Njuho**

South Africa

**Christiana Noestlinger**

Belgium

**Eva Nohlert**

Sweden

**Monica Nolan**

Uganda

**Kurt Nolte**

USA

**Randa Nooh**

Saudi Arabia

**Brian Nordmann**

USA

**Marie Norredam**

Denmark

**Amanda Northcross**

USA

**Ruth Northway**

UK

**Faryle Nothwehr**

USA

**Caitlin Notley**

UK

**Kerri Nottage**

USA

**Thomas Novotny**

USA

**Glen Nowak**

USA

**Mary Patricia Nowalk**

USA

**Krzysztof Nowosielski**

Poland

**Behdin Nowrouzi**

Canada

**Robert Ntozini**

Zimbabwe

**Paula Nunes**

Trinidad and Tobago

**Fred Nuwaha**

Uganda

**Jennifer Nuzzo**

USA

**Batmunkh Nyambat**

Korea, South

**Lotta Nybergh**

Sweden

**Clas-Håkan Nygård**

Finland

**Alan Nyitray**

USA

**Charles Nzioka**

Kenya

**Chikaodili Obidike**

USA

**Ikwo Oboho**

USA

**Jessica Ochalek**

USA

**Annette O'Connor**

USA

**Amos Odiit**

Rwanda

**Angela Odoms-Young**

USA

**Anna Odone**

Italy

**Oluwakemi Odukoya**

Nigeria

**Olumuyiwa Odusanya**

Nigeria

**Maria Ogrzewalska**

Brazil

**Tinuade Ogunlesi**

Nigeria

**Olarinde Ogunmola**

Nigeria

**Oladele Ogunseitan**

USA

**Juhwan Oh**

Korea, South

**Blythe O'Hara**

Australia

**Henrik Ohlsson**

Sweden

**Hirofumi Ohnishi**

Japan

**Masaki Ohsawa**

Japan

**Rie Oka**

Japan

**Tamaki Okabayashi**

Thailand

**Jerry Okal**

Kenya

**Scott Okamoto**

USA

**Oluwakayode Oke**

UK

**Joseph Okeibunor**

Congo

**James Okello**

Uganda

**Jiro Okochi**

Japan

**Chizimuzo Okoli**

USA

**Uduak Okomo**

Gambia

**Regina Oladokun**

Nigeria

**Oluwatosin Olaiya**

USA

**Olaniyi Olayinka**

USA

**Tim Olds**

Australia

**John Oliffe**

Canada

**Aldair Oliveira**

Brazil

**Sérgio Oliveira**

Brazil

**Lemessa Oljira**

Ethiopia

**Samuel Olowookere**

Nigeria

**Brian Olshansky**

USA

**Pia Olsson**

Sweden

**Dana Lee Olstad**

Australia

**Olubode Olufajo**

USA

**Abiodun Oluyomi**

USA

**Sepideh Omidvari**

Iran

**Altan Onat**

Turkey

**Suzanne O'Neill**

USA

**Siobhan O'Neill**

UK

**Ken Ong**

UK

**Maricianah Atieno Onono**

Kenya

**Emerita Opaleye**

Brazil

**Alex Opio**

Uganda

**Alexander Opotowsky**

USA

**Nicoletta Orchi**

Italy

**Andrea Orsi**

Italy

**Adriana Ortega**

Malaysia

**Malina Osman**

Malaysia

**Candice Oster**

Australia

**Esa Osterberg**

Finland

**Ståle Østhus**

Norway

**Rytas Ostrauskas**

Lithuania

**Theresa Osypuk**

USA

**Atsuhiko Ota**

Japan

**Larissa Otero**

Peru

**Alfonso Otero Gonzalez**

Spain

**Nancy Otieno**

Kenya

**Conrad Otterness**

USA

**Kennedy Otwombe**

South Africa

**Katrien Oude Rengerink**

Netherlands

**Nina Cecilie Overby**

Norway

**James Overholser**

USA

**Simon Øverland**

Norway

**Alice Owen**

Australia

**Christopher Owens**

UK

**Ashli Owen-Smith**

USA

**Raymond Ownby**

USA

**Andy Oxman**

Norway

**Abayomi Oyekale**

South Africa

**Koyejo Oyerinde**

USA

**Kotaro Ozasa**

Japan

**Inci Ozgur Ilhan**

Turkey

**Al Ozonoff**

USA

**Markus Paananen**

Finland

**Fernando Pacheco-Torgal**

Portugal

**Christopher Paciorek**

USA

**Carmen Padilla Moledo**

Spain

**Ricardo Pagan**

Spain

**Eva Pagano**

Italy

**Durga Prasad Pahari**

Nepal

**Cristina Palacios**

Puerto Rico

**Wilson Palacios**

USA

**Domingo Palacios-Ceña**

Spain

**Anna Palagyi**

Australia

**Charles John Palenik**

USA

**Claire Palermo**

Australia

**Miranda Pallan**

UK

**António Palmeira**

Portugal

**Jennifer Palmer**

UK

**Shea Palmer**

UK

**Hrefna Palsdottir**

Iceland

**Chen-Wei Pan**

China

**Yi Pan**

USA

**Catalina Panaitescu**

Romania

**Amita Pandey**

India

**Masdalina Pane**

Indonesia

**Ansuman Panigrahi**

India

**Annalisa Pantosti**

Italy

**Maria Papadakaki**

Greece

**Sophia Papadakis**

Canada

**Anastasios Papadimitriou**

Greece

**Evridiki Papastavrou**

Cyprus

**Kristine Pape**

Norway

**Panam Parikh**

Netherlands

**Pasquale Parisi**

Italy

**Yong-Moon Park**

USA

**Min-Hyeon Park**

Korea, South

**Diana Parra**

Brazil

**Manuel Parra**

Chile

**Jose Ruben Parra-Cardona**

USA

**Anne-Maree Parrish**

Australia

**Christopher Parry**

UK

**Sharon Parry**

Australia

**Parisa Parsa**

Iran

**Mallie Paschall**

USA

**Beverley Paterson**

Australia

**Preeti Pathela**

USA

**Arunasalam Pathmeswaran**

Sri Lanka

**Malgorzata Paul**

Poland

**Milena Pavlova**

Netherlands

**Susan Paxton**

Australia

**Raquel Paz Castro**

Switzerland

**Valerie Paz Soldan**

USA

**Gabriela Paz-Bailey**

USA

**Elizabeth Peach**

Australia

**Matthew Pearce**

UK

**Natalie Pearson**

UK

**Anette Pedersen**

Denmark

**Scott Pedersen**

Australia

**Zeljko Pedisic**

Australia

**Ashley Pedler**

Australia

**Monica Peek**

USA

**Nancye May Peel**

Australia

**Nasheeta Peer**

South Africa

**Frank Peinemann**

Germany

**Christine Pellegrini**

USA

**Jennifer Pelletier**

USA

**Karl Peltzer**

South Africa

**Erica Peluso**

Brazil

**Harish Pemde**

India

**Linda Penn**

UK

**Tarra Penney**

UK

**Flavia Pennini**

Chile

**Louisa Peralta**

Australia

**Robert Peralta**

USA

**Gavin Pereira**

USA

**Telmo Pereira**

Portugal

**Marco Peres**

Australia

**Carolina Perez Ferrer**

UK

**Carlos Pérez García-Pando**

USA

**Gastón Perman**

Argentina

**Thomas Perneger**

Switzerland

**Matthew Perri III**

USA

**Joseph Perriens**

Switzerland

**Ivan Perry**

Ireland

**Angela Pesatori**

Italy

**Amber Peterman**

USA

**Remco Peters**

South Africa

**Thomas Peto**

Thailand

**Irene Petritsi Figa Talamanca**

Italy

**Kelley Pettee Gabriel**

USA

**Stefano Petti**

Italy

**Audrey Pettifor**

USA

**Patrizio Pezzotti**

Italy

**Alena M. Pfeil**

Switzerland

**Nittaya Phanuphak**

Thailand

**Mit Philips**

Belgium

**Lisa Phillips**

UK

**Kong Boo Phua**

Singapore

**John Phuka**

Malawi

**Linda Pickle**

USA

**Iris Pigeot**

Germany

**Kustaa Piha**

Finland

**Bettina Piko**

Hungary

**Paul Pilkington**

UK

**Julian David Pillay**

South Africa

**Adriano Pimenta**

Brazil

**Gustavo Duarte Pimentel**

Brazil

**Ilana Pinsky**

Brazil

**Pierluca Piselli**

Italy

**Francesco Pistelli**

Italy

**Andreia Nogueira Pizarro**

Portugal

**Carmine Pizzi**

Italy

**Sandra Plachta-Danielzik**

Germany

**Martin Plöderl**

Austria

**Ronald Plotnikoff**

Australia

**David Plummer**

Australia

**Laura Jean Podewils**

USA

**Pallav Pokhrel**

USA

**Alessandro Polichetti**

Italy

**Benjamin Gregory Polkinghorne**

Australia

**Frank Pollari**

Canada

**Kevin Pollock**

UK

**Sujata Ponappa**

USA

**Alexander Ponizovsky**

Israel

**Amudha Poobalan**

UK

**Nancy Poole**

Canada

**Yong Poovorawan**

Thailand

**Chad Porter**

USA

**David Portnoy**

USA

**Patrick Pössel**

USA

**Ann Post**

Sweden

**Maarten Postma**

Netherlands

**Walker Poston**

USA

**Julio Poterico**

Peru

**Kevin Pottie**

Canada

**Krishna Poudel**

USA

**Mieneke Pouwelse**

Netherlands

**Maura Pozzi**

Italy

**Lorenza Pratali**

Italy

**Frances Press**

Australia

**Garrett Prestage**

Australia

**Jonato Prestes**

Brazil

**Josephine Previte**

Australia

**Kay Price**

Australia

**Malcolm Price**

UK

**William Alex Pridemore**

USA

**Karin Proper**

Netherlands

**Joanne Protheroe**

UK

**Camelia Protopopescu**

France

**Gillian Prue**

UK

**Roman Prymula**

Czech Republic

**Theodora Psaltopoulou**

Greece

**Victor Puac Polanco**

USA

**Eve Puffer**

USA

**Anna Puig-Ribera**

Spain

**Richard Pulsford**

UK

**Vivian Pun**

Hong Kong

**Thandi Puoane**

South Africa

**Pekka Puska**

Finland

**Sandra Putnam**

USA

**Weerasak Putthasri**

Thailand

**Anita Puustjarvi**

Finland

**Yun Qian**

China

**Linda Quan**

USA

**Virginia Quick**

USA

**Evelyn Byrd Quinlivan**

USA

**Rui Quintas**

Italy

**Lieke Raaijmakers**

Netherlands

**Katayoun Rabiei**

Iran

**Labbo Rabiou**

Niger

**George Rachiotis**

Greece

**Sarah Racine**

USA

**Gabriel Rada**

Chile

**Helga Radner**

Austria

**Lisa Rafalson**

USA

**Alberto Raggi**

Italy

**Hina Rahee**

United Arab Emirates

**Chantal Raherison**

France

**Vafa Rahimi-Movaghar**

Iran

**Daniel Rainham**

Canada

**Kristiina Rajaleid**

Sweden

**Mia Rajalin**

Sweden

**Ramakrishnan Ramachandran**

India

**Rohit Ramaswamy**

USA

**Gita Ramjee**

South Africa

**Anu Rammohan**

Australia

**Elisabete Ramos**

Portugal

**Jose Manuel Ramos**

Spain

**Sanjay Rampal**

Malaysia

**Heribert Ramroth**

Germany

**Joakim Ramsberg**

Sweden

**Sally Rankin**

USA

**Geetha Ranmuthugala**

Australia

**Chalapati Rao**

Australia

**Krishna Rao**

India

**Dennis Raphael**

Canada

**Kumanan Rasanathan**

USA

**Parveen Rasheed**

USA

**Giovanna Raso**

Switzerland

**Barbara Rath**

Germany

**Elena Ratschen**

UK

**Garth Rauscher**

USA

**Sofia Ravara**

Portugal

**Ulrike Ravens-Sieberer**

Germany

**Raffaella Ravinetto**

Belgium

**Marissa Raymond-Flesch**

USA

**Nicola Reavley**

Australia

**Matejka Rebolj**

Denmark

**Cassiano Ricardo Rech**

Brazil

**Bernd Rechel**

UK

**Andrea Rechenburg**

Germany

**Elizabeth Reddy**

Tanzania

**Mary Redmayne**

New Zealand

**Julian Reed**

USA

**Aaron Reeves**

UK

**Marina Reeves**

Australia

**Enrique Regidor**

Spain

**David Rehkopf**

USA

**Colin Rehm**

USA

**Esther Reid**

UK

**Natasha Reid**

Australia

**Daniel Reidpath**

Malaysia

**John Reilly**

UK

**Cathrine Reineholm**

Sweden

**Klaus Reinhardt**

UK

**Dominique Reinwand**

Germany

**Anke Reitter**

Germany

**Roseline Remans**

Ethiopia

**Yuan Ren**

China

**Andre Renzaho**

Australia

**Geir K. Resaland**

Norway

**Juan Pablo Rey Lopez**

Brazil

**Mae Lynn Reyes-Rodriguez**

USA

**Heidi Reynolds**

USA

**Mohsen Rezaeian**

Iran

**Leandro Rezende**

Brazil

**Richard Rheingans**

USA

**Karin Rhodes**

USA

**Ryan Rhodes**

Canada

**Sofia Ribeiro**

Portugal

**Elena Ricci**

Italy

**Adam Richards**

USA

**Suzanne Richards**

UK

**Caroline Richardson**

USA

**Lindsey Richardson**

Canada

**Michael Richardson**

USA

**JaNelle Ricks**

USA

**Debra Rickwood**

Australia

**Kate Ridley**

Australia

**Heather Ridolfo**

USA

**Fruhling Rijsdijk**

UK

**Malcolm Riley**

Australia

**Arja Rimpelä**

Finland

**Ning Rintiswati**

Indonesia

**Marit By Rise**

Norway

**Laetitia Rispel**

South Africa

**Chris Rissel**

Australia

**Matthew Ritchey**

USA

**Narjis Rizvi**

Pakistan

**Jose Rizzo**

Brazil

**Bjarne Robberstad**

Norway

**Lorraine Robbins**

USA

**Rachel Roberts**

Australia

**Jennifer Robertson-Wilson**

Canada

**Eric Robinson**

UK

**Mark Robson**

USA

**Lorenzo Rocco**

Italy

**Gustavo Rocha**

Brazil

**Roger Rochat**

USA

**Lance Rodewald**

China

**Dianne Rodger**

Australia

**Ariel Rodriguez**

USA

**Carlos Rodriguez-Galindo**

USA

**Corne Roelen**

Netherlands

**David Roelfs**

USA

**Louise Rohrbach**

USA

**David Rojas-Rueda**

Spain

**Kate Roland**

USA

**John Roll**

USA

**Italia Rolle**

USA

**Josefina Romaguera**

Puerto Rico

**Miguel Roman**

Spain

**Dan Romer**

USA

**Esperanza Romero Estudillo**

Spain

**Mariangela Rondanelli**

Italy

**Claire Rondet**

France

**Olivier Ronveaux**

Burkina Faso

**Robin Room**

Australia

**Irving Rootman**

Canada

**Annina Ropponen**

Finland

**Zeev Rosberger**

Canada

**David Rosen**

USA

**Daniel Rosen**

USA

**Sydney Rosen**

USA

**Mark Rosenberg**

Canada

**Michael Rosenberg**

Australia

**Richard Rosenkranz**

USA

**Judith Rosmalen**

Netherlands

**Craig Ross**

USA

**Michael Ross**

USA

**Simone Ross**

Australia

**Theresa Rossouw**

South Africa

**Berit Rostad**

Norway

**Matteo Rota**

Italy

**Francesco Rotella**

Italy

**Mary Jane Rotheram-Borus**

USA

**Linda Rothman**

Canada

**Heather Rothwell**

UK

**Neneh Rowa-Dewar**

UK

**Marion Rowland**

Ireland

**Gillian Rowlands**

UK

**Emily Rowlinson**

USA

**Debendra Roy**

Bangladesh

**Elizabeth Rubenstone**

USA

**Rima Rudd**

USA

**Kara Rudolph**

USA

**Linda Rudolph**

USA

**Montserrat Rue**

Spain

**George Ruhago**

Norway

**Jean-Bernard Ruidavets**

France

**Chiara Ruini**

Italy

**Godfrey Rukundo**

Uganda

**Andrew Rundle**

USA

**Rosemary Rushmer**

UK

**Tom Russ**

UK

**George Rutherford**

USA

**Guy Rutten**

Netherlands

**Geert Rutten**

Netherlands

**Claire Ryan**

Myanmar

**Agnieszka Rychwalska**

Poland

**Lars Ryden**

Sweden

**Elena Ryder**

Venezuela

**Ramakrishnan Sivasubramanian**

India

**Charumathi Sabanayagam**

Singapore

**Fabio Sabatini**

Italy

**Osman Sabuncuoglu**

Turkey

**Gary Sacks**

Australia

**Ayebo Sadoh**

Nigeria

**Marc Saez**

Spain

**Debasish Saha**

New Zealand

**Shubhayu Saha**

USA

**Rihlat Said-Mohamed**

South Africa

**Pedro F. Saint-Maurice**

USA

**Isao Saito**

Japan

**Rehana Salam**

Pakistan

**Benoit Salanave**

France

**Jordi Salas-Salvadó**

Spain

**Paulo Saldiva**

Brazil

**Sarah Saleem**

Pakistan

**Maria Cristina Salfa**

Italy

**Muhannad Salih**

Malaysia

**Andrea Salis**

USA

**Antti Saloniemi**

Finland

**Ferdinand Salonna**

Czech Republic

**Juha Saltevo**

Finland

**Sonia Saluja**

Australia

**Taraz Samandari**

USA

**Oddrun Samdal**

Norway

**Asa Samuelsson**

Sweden

**Hiromi Sanada**

Japan

**Zila Sanchez**

Brazil

**Kristy Sanderson**

Australia

**Maureen Sanderson**

USA

**Paul Sanderson**

UK

**Carine Sangaleti**

Brazil

**Padmaja Sankaridurg**

Australia

**Aboukabary Sanou**

Canada

**Silvina Santana**

Portugal

**John Santelli**

USA

**Jaime Sapag**

Chile

**Valeria Saraceni**

Brazil

**Fred Sarfo**

Ghana

**Mary Saridi**

Greece

**Anna Sarkadi**

Sweden

**Mohamadi Sarkar**

USA

**Olga Sarmiento**

Colombia

**Avina Sarna**

India

**Ilhan Satman**

Turkey

**Radhanath Satpathy**

India

**Kamala Kanta Satpathy**

India

**Travis Saunders**

Canada

**Jennifer Savage**

USA

**Valeria Savasi**

Italy

**Naomi Saville**

UK

**Susumu Sawada**

Japan

**Peter Scarborough**

UK

**Brooke Scelza**

USA

**Ingmar Schäfer**

Germany

**Dena Schanzer**

Canada

**Gillian Schauer**

USA

**Susan Scheer**

USA

**Taneisha Scheuermann**

USA

**Barbara Schillo**

USA

**Christian Schindler**

Switzerland

**Jasper Schipperijn**

Denmark

**Gerard M Schippers**

Netherlands

**Robert Schlack**

Germany

**Carla Schlesinger**

Australia

**Barbara Schneider**

Germany

**Francine Schneider**

Netherlands

**Daniel D. Schnitzlein**

Germany

**Inga Schnuerer**

Germany

**Nancy Schoenberg**

USA

**Shaun Scholes**

UK

**Jon Schommer**

USA

**Gerhard Schön**

Germany

**James Schreiber**

USA

**Helmut Schroder**

Spain

**Julian Schulze zur Wiesch**

Germany

**Merel Schuring**

Netherlands

**Norbert Georg Schwarz**

Germany

**Ralf Schwarzer**

Germany

**Jürgen Schweikart**

Germany

**Lukas Schwingshackl**

Austria

**John Scott**

Australia

**Paul Scott**

USA

**Robert Scragg**

New Zealand

**Mark Scudder**

USA

**André Seabra**

Portugal

**Holly Seale**

Australia

**Bernadette Sebar**

Australia

**Jan Seghers**

Belgium

**Victor Segura-Jimenez**

Spain

**Susan Seibold-Simpson**

USA

**Juliet Sekandi**

Uganda

**Silvia Selinski**

Germany

**Chloe Sellwood**

UK

**Joseph Sempa**

Uganda

**Maya Semrau**

UK

**Paramita Sengupta**

India

**Gunes Senol**

Turkey

**Meghan Senso**

USA

**Mariane Sentenac**

France

**Diego Serraino**

Italy

**Veerasamy Seshiah**

India

**Dinesh Sethi**

Denmark

**Vivian Nguyan Shaahu**

Nigeria

**Shobha Shah**

India

**Nazma Shaheen**

Bangladesh

**Mirja Mohammad Shahjamal**

Bangladesh

**Masoud Shamaei**

Iran

**Akhawri Shankar**

India

**Christopher Shanley**

Australia

**Frank Shann**

Australia

**Saida Sharapova**

USA

**Mamak Shariat**

Iran

**Suleiman Sharif**

United Arab Emirates

**Kamal Sharma**

India

**Vartika Sharma**

India

**Norman Sharpe**

New Zealand

**Lisa Sharwood**

Australia

**Benjamin Shaw**

USA

**Caroline Shaw**

New Zealand

**Kate Shaw**

USA

**Aubrey Sheiham**

UK

**Brent Shelton**

USA

**Rachel Shelton**

USA

**Sheela Shenoi**

USA

**Shweta Shenoy**

India

**Bhupendra Sheoran**

USA

**Roya Sherafat Kazemzadeh**

USA

**Jessica Sheringham**

UK

**Recinda Sherman**

USA

**Ju-Fang Shi**

China

**Yuyan Shi**

USA

**Zumin Shi**

Australia

**Ai Shibata**

Japan

**Ruth Shim**

USA

**Techalew Shimelis**

Ethiopia

**Dong Wook Shin**

Korea, South

**Nathan Shippee**

USA

**Yehuda Shoenfeld**

Israel

**Steve Shoptaw**

USA

**Camille Short**

Australia

**Niamh Shortt**

UK

**Ashish Chandra Shrestha**

Nepal

**Lenka Shriver**

USA

**Sharvari Shukla**

India

**Tianmei Si**

China

**Kamran Siddiqi**

UK

**Estelle Monique Sidze**

Kenya

**Aaron Siegler**

USA

**Johannes Siegrist**

Germany

**Alicja Sieminska**

Poland

**Madeleine Sigman-Grant**

USA

**Erik Sigmund**

Czech Republic

**Jelle Sijtsema**

Netherlands

**Claudia Sikorski**

Germany

**Sheetal Silal**

South Africa

**Sven Silburn**

Australia

**Carlos Silva**

Brazil

**Maria Laura Silva**

France

**Paula Silva**

Portugal

**Giulia Silvestrini**

Italy

**David Simmons**

UK

**Francisco Simões**

Portugal

**Melissa Simon**

USA

**Dorien Simons**

Belgium

**Jacob Simonsen**

Denmark

**Venka Simovska**

Denmark

**Irit Sinai**

USA

**David Sinclair**

UK

**Nandita Singh**

Sweden

**Tushar Singh**

USA

**Mahmoud Sirdah**

Palestinian Territory

**Suttajit Sirijit**

Thailand

**Susan Sisson**

USA

**Anna Siukola**

Finland

**Agneta Sjöberg**

Sweden

**Kelly Skinner**

Canada

**Marit Skogstad**

Norway

**Emma Slaymaker**

UK

**Terry Slevin**

Australia

**Samy Slimani**

Algeria

**Fiona Smaill**

Canada

**Dylan Small**

USA

**Nada Smigic**

Serbia

**Eline Smit**

Netherlands

**Ellen Smit**

USA

**Amber Smith**

USA

**Andrea Smith**

Australia

**Ben Smith**

Australia

**Claire Smith**

New Zealand

**Ingrid Smith**

Norway

**Kylie Smith**

Australia

**Lee Smith**

UK

**Lisa Smith**

USA

**Patricia Smith**

Canada

**Andy Smith**

UK

**Amy Smoyer**

USA

**John Snelgrove**

Canada

**Robert William Snow**

Kenya

**Patricia Soarez**

Brazil

**Helene L Soberg**

Norway

**Diana Sobieraj**

USA

**Morten Sodemann**

Denmark

**Woosung Sohn**

USA

**Marit Solbjør**

Norway

**Maria Giuliana Solinas**

Italy

**Samir Soneji**

USA

**Pam Sonnenberg**

UK

**Heng Sopheab**

Cambodia

**Elpidoforos Soteriades**

Cyprus

**Giovanni Sotgiu**

Italy

**Daniela Sotres-Alvarez**

USA

**Hugo Sousa**

Portugal

**Alex Souza**

Brazil

**Ireneous N. Soyiri**

Malaysia

**Kaan Sözmen**

Turkey

**Fredrik Spak**

Sweden

**Ross Sparks**

Australia

**Carina Sparud-Lundin**

Sweden

**Anne Spaulding**

USA

**Rick Speare**

Australia

**John Spence**

Canada

**Niko Speybroeck**

Belgium

**Elodie Speyer**

France

**Matthew Spittal**

Australia

**Waney Squier**

UK

**Hazel Squires**

UK

**Jorge Srabstein**

USA

**Chandrashekhar Sreeramareddy**

Nepal

**Stella Stabouli**

Greece

**Steven Stack**

USA

**Marietta Stadler**

UK

**Mai Stafford**

UK

**Martin Stafström**

Sweden

**Amanda Staiano**

USA

**Mindaugas Stankunas**

Lithuania

**Rebecca Stanley**

Australia

**Maja Stanojevic**

Serbia

**Chris Staples**

Australia

**Anita Star**

Australia

**Anke Steckelberg**

Germany

**Jim van Steenbergen**

Netherlands

**Jostein Steene-Johannessen**

Norway

**Ivan Steenstra**

Canada

**Matthew Stefanak**

USA

**Aryeh Stein**

USA

**Catherine Stein**

USA

**Juergen Steinacker**

Germany

**Riley Steiner**

USA

**Peter Steinmann**

Switzerland

**Sarit Steinmetz**

Israel

**Aslak Steinsbekk**

Norway

**Marlene Stenbacka**

Sweden

**Craig Stephen**

Canada

**Nathan Stephens**

UK

**Christopher Stevenson**

Australia

**Leah Stevenson**

Australia

**Alistair Stewart**

New Zealand

**Diana Stewart**

USA

**Joanna Stewart**

New Zealand

**Jennifer Stewart Williams**

Australia

**Andrew Stickley**

Sweden

**Henk Stipdonk**

Netherlands

**Christian Stock**

Germany

**Tim Stockwell**

Canada

**Steven Stoddard**

USA

**Barbara Stoecker**

USA

**Manfred Stommel**

USA

**Lee Stoner**

New Zealand

**Jodi Stookey**

USA

**Gareth Stratton**

UK

**William Strawbridge**

USA

**Lyndall Strazdins**

Australia

**Claudia Strugnell**

Australia

**Heather Stuart**

Canada

**James Stuart**

UK

**Matthew Stults-Kolehmainen**

USA

**Jason Su**

USA

**Hong Su**

China

**Pushp Kamal Subedi**

Nepal

**Takemi Sugiyama**

Australia

**Zhixian Sui**

Australia

**Kadri Suija**

Finland

**Ernest Sumaili**

Congo, Democratic Republic

**Carolyn Summerbell**

UK

**Colin Sumpter**

UK

**Peifang Sun**

USA

**Ye-Huan Sun**

China

**Johanne Sundby**

Norway

**Karin Sundström**

Sweden

**Bruno Sunguya**

Japan

**Tarja Suominen**

Finland

**Johari Surin**

Malaysia

**Fernanda G C Surita**

Brazil

**Michele Sutherland**

Australia

**Etsuji Suzuki**

Japan

**Kohta Suzuki**

Japan

**Motoi Suzuki**

Japan

**Igor Svab**

Slovenia

**Margrét Svavarsdóttir**

Norway

**Jannet Svensson**

Denmark

**Katarina Swahnberg**

Sweden

**Waleed Sweileh**

Palestinian Territory

**Ilse Swinkels**

Netherlands

**Girija Syamlal**

USA

**Lene Symes**

USA

**Jennifer Syvertsen**

USA

**Lisa Szatkowski**

UK

**Adam Szpiro**

USA

**Alex Szubert**

UK

**Henri Taanila**

Finland

**Theonie Tacticos**

Australia

**Bewket Tadesse**

Ethiopia

**Dirk Taeger**

Germany

**Angela Taft**

Australia

**Husref Tahirovic**

Bosnia and Herzegovina

**Marc Christian Tahita**

Burkina Faso

**Richard Tahtinen**

Iceland

**Marko Tainio**

UK

**Daisuke Takagi**

Japan

**Jiro Takaki**

Japan

**Soshi Takao**

Japan

**Simbarashe Takuva**

South Africa

**Evelyn Talbott**

USA

**Yin On Yvonne Tam**

USA

**Teresa Tamayo**

Germany

**Sietske Tamminga**

Netherlands

**Abdonas Tamosiunas**

Lithuania

**Kosuke Tamura**

USA

**Andy Tan**

USA

**Darrell Tan**

Canada

**Maw Pin Tan**

Malaysia

**Junko Tanaka**

Japan

**Stephanie Tanamas**

Australia

**Fei Tang**

China

**Shinichi Tanihara**

Japan

**Susan Tanner**

USA

**Elisabetta Tanzi**

Italy

**Jose Tapia Granados**

USA

**David Tappin**

UK

**Tyna Taskila**

UK

**Marie-Pierre Tavolacci**

France

**Paula Tavrow**

USA

**Suthira Taychakhoonavudh**

Thailand

**Biruhalem Taye**

Ethiopia

**Amy Taylor**

UK

**David Taylor**

USA

**Saskia te Velde**

Netherlands

**Karl-Gunter Technau**

South Africa

**Alison Tedstone**

UK

**Conor Teljeur**

Ireland

**Charles Teller**

USA

**Michelle Templeton**

UK

**Gill ten Hoor**

Netherlands

**Laura Ternent**

UK

**Yvonne Terry-McElrath**

USA

**Katrien Tersago**

Belgium

**Kay Teschke**

Canada

**Markos Tesfaye**

Ethiopia

**Christian Teter**

USA

**Garry Tew**

UK

**Nai-peng Tey**

Malaysia

**Lucy Thairu**

USA

**Maniam Thambu**

Malaysia

**Dag Steinar Thelle**

Norway

**Markus Themessl-Huber**

UK

**Cecilie Thogersen-Ntoumani**

Australia

**Grace Thoithi**

Kenya

**Brett Thombs**

Canada

**RC Andrew Thompson**

Australia

**Deborah Thompson**

UK

**Lisa Thompson**

USA

**Michael Thompson**

USA

**Jo Thompson Coon**

UK

**Cynthia Thomson**

USA

**George Thomson**

New Zealand

**Hilary Thomson**

UK

**William Murray Thomson**

New Zealand

**Ingibjörg Thorisdottir**

Iceland

**Bruce Thorley**

Australia

**Anne Thorndike**

USA

**Lukar Thornton**

Australia

**Anne Marie Thow**

Australia

**Wilfreda E. Thurston**

Canada

**Yuan Tian**

Singapore

**James Tibenderana**

Uganda

**Hong Van Tieu**

USA

**Stelios Tigas**

Greece

**Marelign Tilahun**

Ethiopia

**Elizabeth Tilley**

Switzerland

**Therese Tillin**

UK

**Lada Timotijevic**

UK

**Norman Tinanoff**

USA

**Christiana Titaley**

Indonesia

**Sylvia Titze**

Austria

**Susanna Toivanen**

Sweden

**Mariam Tokhi**

Australia

**Jigsa Tola**

USA

**Marcus Tolentino Silva**

Brazil

**Luciano Toma**

Italy

**Venera Tomaselli**

Italy

**Martin Tondel**

Sweden

**Kwok Kit Tong**

Macao

**Van Tong**

USA

**Rebecca Tooher**

Australia

**Margareta Torgén**

Sweden

**Nuria Torner**

Spain

**Belen Torondel**

UK

**Kwasi Torpey**

Nigeria

**Elizabeth Torrone**

USA

**Guillermo Tortolero**

Puerto Rico

**Igor Toskin**

Switzerland

**Alison Tovar**

USA

**Loraine Townsend**

South Africa

**Alberto Tozzi**

Italy

**Irene Tramacere**

Italy

**Duong Tran**

Australia

**Thanh Tran**

USA

**Stewart Trost**

Australia

**Elisa Trucco**

USA

**Jack Tsai**

USA

**Jennifer Tsai**

USA

**Boldtsetseg Tserenpuntsag**

USA

**Cleon Tsimbos**

Greece

**Zoi Tsimtsiou**

Greece

**Kelvin Tsoi**

Hong Kong

**Kanami Tsuno**

Japan

**Kenji Tsunoda**

Japan

**Nguyen Tuan**

Viet Nam

**Sandy Tubeuf**

UK

**Adriana Tucci**

Brazil

**Graeme Tucker**

Australia

**Joseph Tucker**

China

**Patricia Tucker**

Canada

**Reginald Tucker-Seeley**

USA

**Terry Tudor**

UK

**Ashleigh Tuite**

Canada

**Josep A. Tur**

Spain

**Gustavo Turecki**

Canada

**Rick Turner**

USA

**Graham Turpin**

UK

**Gavin Turrell**

Australia

**Konstantinos Tziomalos**

Greece

**Zia Ul Haq**

Pakistan

**Shahid Ullah**

Australia

**Bernhard Ultsch**

Germany

**M. Renee Umstattd Meyer**

USA

**Leanne Unicomb**

Bangladesh

**Lianne Urada**

USA

**Sebastien Urben**

Switzerland

**Sorin Ursoniu**

Romania

**Richard Uwakwe**

Nigeria

**Isabelle Vachier**

France

**Carmina Valle**

USA

**Natalia Vallianou**

Greece

**Mahara Valverde**

Mexico

**Patricia van Assema**

Netherlands

**Ed van Beeck**

Netherlands

**Jantien van Berkel**

Netherlands

**Paul Van Buynder**

Canada

**Jelle Van Cauwenberg**

Belgium

**Bart van den Borne**

Netherlands

**Hendrik van den Bussche**

Germany

**Iris van der Heide**

Netherlands

**Johan Hubert André Van der Heyden**

Belgium

**Wim van der Hoek**

Netherlands

**Marianne A.B. van der Sande**

Netherlands

**Alike van der Velden**

Netherlands

**Marieke J. van der Werf**

Netherlands

**Bernard Van der Zeijst**

Netherlands

**Martin Van Dijk**

Netherlands

**Paula van Dommelen**

Netherlands

**Dave Van Kann**

Netherlands

**Ellen van Kleef**

Netherlands

**Frank van Lenthe**

Netherlands

**Geneviève van Liere**

Netherlands

**Wendy Van Lippevelde**

Belgium

**Femke Van Nassau**

Netherlands

**Sjan-Mari van Niekerk**

South Africa

**Sandra Helena van Oostrom**

Netherlands

**Mireille van Poppel**

Netherlands

**Annelies Van Rie**

USA

**Ronan Van Rossem**

Belgium

**Esther van Sluijs**

UK

**Angela van Tilborg**

Netherlands

**Lex van Velsen**

Netherlands

**Nicholas Van Wagoner**

USA

**Edwin van Wijngaarden**

USA

**Gert van Zyl**

South Africa

**Johan Martin van Zyl**

South Africa

**Nicola Vanacore**

Italy

**Corneel Vandelanotte**

Australia

**Kerry Vander Ploeg**

Canada

**Christophe Vanroelen**

Belgium

**Kiruba Sankar Varadharajan**

India

**Constantine Vardavas**

USA

**Inge Varekamp**

Netherlands

**Veronica Varela Mato**

UK

**Maria Ines Varela-Silva**

UK

**Constanza Vargas Parada**

Chile

**Beatriz Vautin**

USA

**Lennert Veerman**

Australia

**Antonio Vela-Bueno**

Spain

**Martha Velandia**

USA

**Anoop Velayudhan**

India

**Louiza Velentzis**

Australia

**Sudhir Venkatesan**

UK

**Homer Venters**

USA

**Marcos Vera-Hernandez**

UK

**Stephane Verguet**

USA

**Peter Verhaak**

Netherlands

**Aukje Verhoeven**

Netherlands

**Heleen Vermandere**

Belgium

**Willemijn Mathilda Vermeer**

Netherlands

**Michel Vernay**

France

**Eleni Verykouki**

Greece

**Fenicia Vescio**

Italy

**Umberto Vespasiani-Gentilucci**

Italy

**Christina Victor**

UK

**Helen Vidgen**

Australia

**Jeffrey Vietri**

Italy

**Alessandro Viggiano**

Italy

**Federica Vigna-Taglianti**

Italy

**Froydis N Vik**

Norway

**Joan R Villalbí**

Spain

**Ester Villalonga-Olives**

Germany

**Anthony Villani**

Australia

**Karen Villanueva**

Australia

**Alberto Villarejo**

Spain

**Marianna Virtanen**

Finland

**Bilkis Vissandjee**

Canada

**Tommy Visscher**

Netherlands

**Anand Viswanathan**

India

**Hristina Vlajinac**

Serbia

**Helene Voeten**

Netherlands

**Olaf von dem Knesebeck**

Germany

**Michael Von Korff**

USA

**Sophie von Stumm**

UK

**Sirenda Vong**

Cambodia

**Carmen Voogt**

Netherlands

**Theo Vos**

USA

**Alexander Vu**

USA

**Anne Vuillemin**

France

**Bea Vuylsteke**

Belgium

**Juddy Wachira**

Kenya

**Olivia Wackowski**

USA

**Timothy Wade**

USA

**Julia Wager**

Germany

**Jennifer Wagman**

USA

**Anjuli Wagner**

USA

**Glenn Wagner**

USA

**Kolawole Wahab**

Nigeria

**Hayfaa Wahabi**

Sudan

**Morten Wahrendorf**

Germany

**Linda Waite**

USA

**Dawn-Marie Walker**

UK

**Lorraine Walker**

USA

**Simon Walker**

UK

**Jennifer Walker**

Australia

**Martin Wall**

New Zealand

**Dean Wallace**

UK

**Lance Waller**

USA

**Kate Walsh**

USA

**Kim Walsh-Childers**

USA

**Judd Walson**

USA

**Richard Wamai**

USA

**Thomas Wan**

USA

**Xia Wan**

China

**Handan Wand**

Australia

**Gustavo Wandalsen**

Brazil

**Per Wandell**

Sweden

**Chong-Wen Wang**

Hong Kong

**Changlu Wang**

USA

**Haiyan Wang**

USA

**JianLi Wang**

Canada

**Jianming Wang**

China

**Man Ping Wang**

Hong Kong

**Qun Wang**

China

**Haibo Wang**

China

**Jinfeng Wang**

China

**Yujie Wang**

USA

**Rhoda Wanyenze**

Uganda

**Dianne Ward**

USA

**Paul Ward**

Australia

**Nasir Warfa**

UK

**Saman Warnakulasuriya**

UK

**John Warren**

USA

**Hunniya Waseem**

Pakistan

**Belaynew Wasie**

Ethiopia

**Rosanna Watowicz**

USA

**Don Webber**

UK

**Allison Webel**

USA

**Ellerie Weber**

USA

**Marianne Weber**

Australia

**Mary Beth Weber**

USA

**E Kipling Webster**

USA

**Premila Webster**

UK

**Stevan Weine**

USA

**William Weiss**

USA

**Jennifer Weiss**

USA

**Emma Weitkamp**

UK

**James R. Welch**

Brazil

**Lyndal Wellard**

Australia

**Heini Wennman**

Finland

**Heiman Wertheim**

Viet Nam

**Peter J.M. Westerholm**

Sweden

**Klaas Westerterp**

Netherlands

**Philip Westgate**

USA

**Simone Weyers**

Germany

**David Wheeler**

UK

**Ana Wheelock**

UK

**Elizabeth Whipple**

USA

**Daniel Whitaker**

USA

**Kellee White**

USA

**Lisa White**

Thailand

**Geoffrey Whitfield**

USA

**Matthias Wicki**

Switzerland

**Priya Wickramaratne**

USA

**Douglas Wiebe**

USA

**Frank Wieber**

Germany

**John Wiecha**

USA

**Jannah Wigle**

UK

**Frans Willekens**

Germany

**Gonneke Willemsen**

Netherlands

**Joshua Willey**

USA

**Craig Williams**

UK

**David Williams**

UK

**Gary Williams**

Australia

**John Williams**

UK

**Julianne Williams**

UK

**Marie Williams**

Australia

**Mark Williams**

USA

**Paige Williams**

USA

**Simon Williams**

USA

**Gordon Willis**

USA

**Wendy Wills**

UK

**Amanda Wilson**

Australia

**Caroline Wilson**

UK

**Hannah Wilson**

USA

**Anna Wilson**

USA

**Helen Winefield**

Australia

**William Winfrey**

USA

**Godfrey Woelk**

USA

**Daniel Wojdyla**

Argentina

**Mirkuzie Woldie**

Ethiopia

**Richard Wolitski**

USA

**Frank Wong**

Uzbekistan

**Paul W.C. Wong**

Hong Kong

**Ruey-Hong Wong**

Taiwan

**Wai Pong Wong**

Singapore

**Jean Woo**

Hong Kong

**Kam Sang Woo**

China

**James Wood**

UK

**Fiona Wood**

UK

**James Woodcocck**

UK

**Richard Woodman**

Australia

**Rebecca Woodruff**

USA

**Cynthia Woodsong**

South Africa

**Alistair Woodward**

New Zealand

**Sarah Woolf-King**

USA

**Anne Woolridge**

UK

**Alemayehu Worku**

Ethiopia

**Anthony Worsley**

Australia

**Edwin Wouters**

Belgium

**Adugna Woyessa**

Ethiopia

**Wendy Wrieden**

UK

**Alison Wright**

UK

**Jim Wright**

UK

**Anise M. S. Wu**

China

**Elwin Wu**

USA

**Qi Wu**

UK

**Jingwei Wu**

USA

**Yangfeng Wu**

China

**Zunyou Wu**

China

**Luh Putu Lila Wulandari**

Indonesia

**Huiyun Xiang**

USA

**Biao Xu**

China

**Fei Xu**

China

**Wanghong Xu**

China

**Haiwen Xu**

China

**Fuzhong Xue**

China

**Zaida Estela Yadon**

Brazil

**Estifanos Yalew**

Ethiopia

**Eileen Yam**

USA

**Yongping Yan**

China

**Joshua Yang**

USA

**Tingzhong Yang**

China

**Xiaolin Yang**

Finland

**Lin Yang**

USA

**Yanjie Yang**

China

**George Yannis**

Greece

**Ahmed Yaogo**

France

**Ohid Yaqub**

UK

**Kristin Yarris**

USA

**Zimri Yaseen**

USA

**Annalee Yassi**

Canada

**Nasser Yassin**

Lebanon

**Kawahata Yasuko**

Japan

**Akitomo Yasunaga**

Japan

**Alfred Yawson**

Ghana

**Jan Fekke Ybema**

Netherlands

**Shaodong Ye**

China

**Mahbuba Yeasmin**

Australia

**Hung-Wen Yeh**

USA

**Adoke Yeka**

Uganda

**Wendemagegn Enbiale Yeshaneh**

Ethiopia

**C. Albert Yeung**

UK

**Shirley Yeung**

UK

**Edwina Yeung**

USA

**Christopher Yilgwan**

Nigeria

**Solomon A Yimer**

Norway

**Zheng Yin**

UK

**Gui-shuang Ying**

USA

**Oded Yitschaky**

Israel

**Renata Yokota**

Belgium

**Koji Yonemoto**

Japan

**Hua Yong**

Australia

**Kouichi Yoshimasu**

Japan

**Marcel Yotebieng**

USA

**April Young**

USA

**Ian Young**

Canada

**Honor Young**

UK

**Kathryn Yount**

USA

**Doris Yu**

Hong Kong

**Jinming Yu**

China

**Xue Qin Yu**

Australia

**Catherine Yu**

Canada

**Guo-Pei Yu**

China

**Hongjie Yu**

China

**Jiao Yumei**

China

**Fakir Yunus**

Bangladesh

**Rafiu Yusuf**

Nigeria

**Apolinaras Zaborskis**

Lithuania

**Matthew Zack**

USA

**Jennifer Zambriski**

USA

**Susan Zbikowski**

USA

**Hajo Zeeb**

Germany

**Zeyede Zeleke**

Ethiopia

**April Zeoli**

USA

**Elsa Zerbini**

Argentina

**Jianduan Zhang**

China

**Tiejun Zhang**

China

**Xi Zhang**

China

**Xinping Zhang**

China

**Yuan Zhang**

USA

**Rui Zhao**

China

**Lijun Zheng**

China

**Pinpin Zheng**

China

**Yanbing Zhou**

USA

**Haidong Zhu**

USA

**Motao Zhu**

USA

**Shu-Hong Zhu**

USA

**Yong-Guan Zhu**

China

**Bao-Ping Zhu**

USA

**Jinliang Zhu**

Denmark

**Qin Zhuo**

China

**Frederick Zimmerman**

USA

**Frank Zimmermann-Viehoff**

Germany

**Denis Zmirou-Navier**

France

**Farzaneh Zolala**

Iran

**Huachun Zou**

Australia

**William Zule**

USA

**Isaac Zulu**

USA

**Li Zuo**

USA

